# Advanced Bioelectrical Signal Processing Methods: Past, Present and Future Approach—Part II: Brain Signals

**DOI:** 10.3390/s21196343

**Published:** 2021-09-23

**Authors:** Radek Martinek, Martina Ladrova, Michaela Sidikova, Rene Jaros, Khosrow Behbehani, Radana Kahankova, Aleksandra Kawala-Sterniuk

**Affiliations:** 1 Department of Cybernetics and Biomedical Engineering, VSB-Technical University Ostrava—FEECS, 708 00 Ostrava-Poruba, Czech Republic; martina.ladrova@vsb.cz (M.L.); michaela.sidikova@vsb.cz (M.S.); rene.jaros@vsb.cz (R.J.); radana.kahankova@vsb.cz (R.K.); 2 College of Engineering, The University of Texas in Arlington, Arlington, TX 76019, USA; kb@uta.edu; 3 Faculty of Electrical Engineering, Automatic Control and Informatics, Opole University of Technology, 45-758 Opole, Poland

**Keywords:** brain signals, signal processing methods, electroencephalography, electrocorticography, bioelectrical signals

## Abstract

As it was mentioned in the previous part of this work (Part I)—the advanced signal processing methods are one of the quickest and the most dynamically developing scientific areas of biomedical engineering with their increasing usage in current clinical practice. In this paper, which is a Part II work—various innovative methods for the analysis of brain bioelectrical signals were presented and compared. It also describes both classical and advanced approaches for noise contamination removal such as among the others digital adaptive and non-adaptive filtering, signal decomposition methods based on blind source separation, and wavelet transform.

## 1. Introduction

The most complex “computer” in the world is the human brain. Despite numerous attempts, no one has ever managed to completely model its overall operation [1,2,3]. It consists of as many as 100 billion neurons, and each neuron can create as many as 10,000 synaptic connections with other nerve cells. The brain is jelly-like in consistency because it is mostly water [4]. Its mass accounts for about 2% of the total human body mass and consumes 20% of the energy produced by the body. The very process of thinking is based on electricity and chemistry—in an active state, the brain produces energy of about 25 W, which is enough to light up a light bulb. At the moment, despite so much research and such extensive knowledge about the brain, there are still many unknowns [2].

The recent growing interest in the analysis of biomedical data developed a lot of innovations and advances. These created opportunities to develop and apply novel algorithms for more sophisticated and effective noise decontamination of biological signals to obtain clinically useful information—also in real-time. These techniques include, for example, improved classical non-adaptive or adaptive filters, signal decomposition methods, or hybrid algorithms.

Biomedical data (in particular brain signals) are very difficult from an analytical point of view, mainly due to their non-stationary nature and low amplitude and low-frequency range. Moreover, these signals are often noisy and contaminated with various artifacts, which negatively affects their potential processing utility [5,6].

This paper provides an extensive review of the latest methods applied for the processing of brain signals. Herein, the most popular methods were summarized and those most efficient were presented in detail.

## 2. Electroencephalography

**Figure 1:**
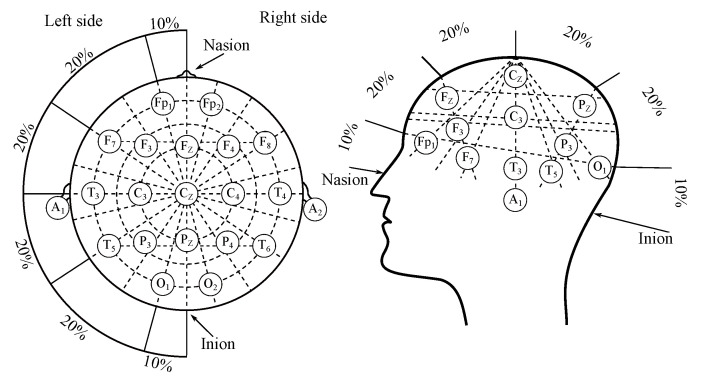
The EEG electrodes placement system “10–20”.

Electroencephalography (EEG) is a diagnostic method, enabling measurement and recording of the electrical activity of the brain [7,8,9]. The measurement of the EEG can be classified as either invasive or non-invasive, depending on surgical intervention necessity. The non-invasive measurement method is based on electrodes placed on the scalp, in accordance with the “10–20” system (or its modifications such as “10–10” or “10–5”) as illustrated in Figure 1 [10,11]. The invasive recordings require e.g., needle electrodes [11].

**Figure 2:**
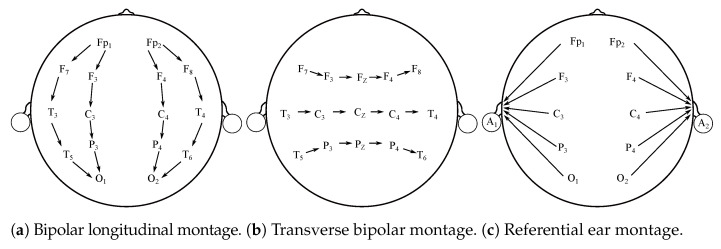
Various EEG montages.

Besides typical “10–20” EEG montages, and their variation, it is possible to differentiate other electrodes’ placement systems [12,13]. The montages can be bipolar, referential, common average, or Laplacian [12]. These montages have various potential clinical implementations and are used in clinical applications mostly. The main purpose for seeking alternatives to the typical 10–20 system is the necessity for finding particular abnormalities. They are also frequently applied in Epilepsy and sleep-related studies [12,14]. Figure 2 illustrates the above-mentioned alternative montages.

The EEG is usually applied for the purpose of neurological and psychological disorders or epileptic seizures examination. It is also used for monitoring various stages of sleep. Another implementation of electroencephalography is brain interaction with external environments in the form of Brain-Computer Interface (BCI) systems [5,15,16,17,18,19].

**Figure 3:**
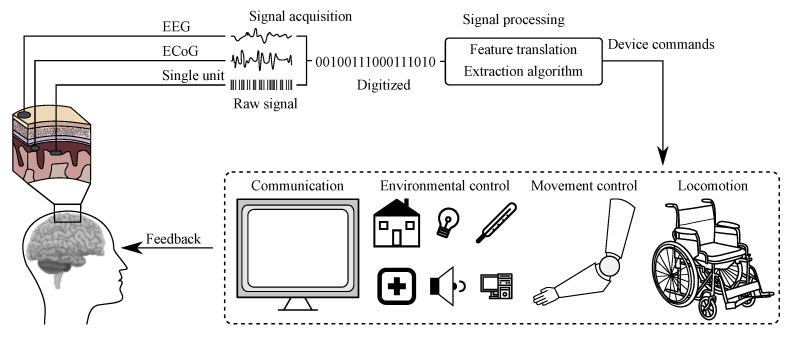
Scheme of a BCI system [20].

The BCI history started already in the 1970s of the 20th century and the rapid development of these systems has led to really efficient communication between the human brain and computers [20]. The BCI refers to a system, which measures and applies signals obtained from the central nervous system. Thus, according to this definition, other Human-Machine Systems based on biomedical data, such as inter alia voice- or and muscle-activated systems, cannot be considered as BCI [20,21]. In these (BCI) systems the signal acquired from the brain is being analyzed and translated into appropriate commands, which may allow full or partial replacement of external devices, such as computer keyboard, mouse, or joystick to perform an action [21]. Scheme illustrating the work of a typical BCI system is presented in Figure 3 [20].

As it was mentioned above 10–20—the clinical EEG applies an international system of the surface electrodes placement—“10–20”, which was illustrated with Figure 1 [10,11]. The system is derived from the distance between the electrodes, where the “10” and “20” values correspond with the 10 and 20% values of the distance between “mastoids” and between nasion and inion, which are the extreme points. “10–20” system consists of 21 electrodes in total, however, only 19 are placed on the surface of the scalp, where the 2 are usually applied on earlobes as reference channels. There are also variations of this system, and these were mentioned above, where the distance was decreased to 10 or 5% to place more electrodes [10,11,22].

For the clinical measurement of sleep usually from 8 to 21 channels are applied, while scientific activities may require up to 256 channels placed on the scalp surface [22,23]. The usual length of recording is around 20 min, but the sleep stages tracking or the diagnosis of epilepsy may take several hours or days [24,25].

The amplitude of the signal is very low, reaching values of around 100 μV. The most commonly applied sampling frequency for the EEG signal is 512 Hz, for EP (Evoked Potential) up to 6 kHz. The frequency range of the EEG signals is up to 80 Hz, but for routine diagnostic testing a range of 0.5–50 Hz [5,16,26,27,28].

### 2.1. EEG Recordings

**Figure 4:**
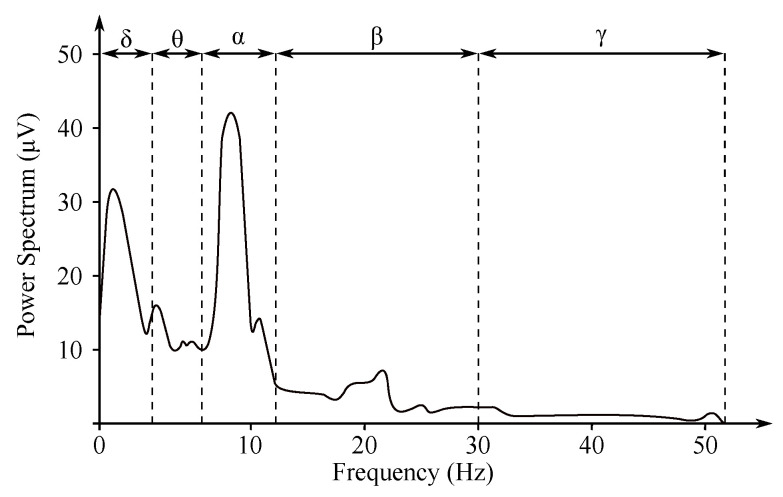
EEG frequency spectrum and its frequency bands [31].

The non-invasive EEG recordings are recorded from the electrodes within the below listed ranges, which correspond with the individual frequency domains (see Figure 4) [5,29,30]:

- **Delta** rhythm with a frequency of 0.5≤f≤4 Hz is a symptom of deeper stages of sleep. Its occurrence can also be observed in the newborn EEG recordings;
- **Theta** rhythm with a frequency of 4≤f≤8 Hz is found in the initial stages of sleep;
- **Alpha** rhythm with a frequency of 8≤f≤13 Hz is the main manifestation of the resting brain activity. The highest values are obtained when during the so-called relaxed alertness;
- **Beta** rhythm with a frequency of 13≤f≤30 Hz is present in nervous or anxious subjects. The amplitude does not exceed 20 μV. It is associated with higher cognitive functions;
- **Gamma** rhythm with a frequency of 30≤f≤80 Hz is associated with high cognitive functions as a response to various stimuli.

**Figure 5:**
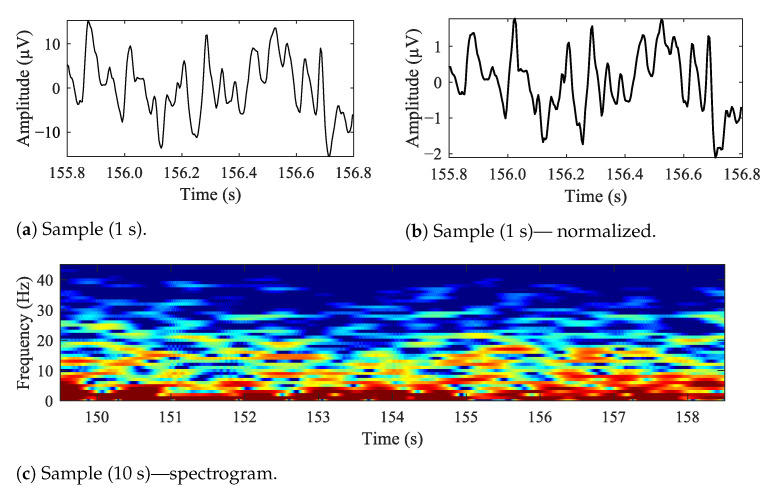
Sample EEG data recorded from ‘F3–C3’ location.

In Figure 5, sample EEG data is presented. In Figure 5a is a 1 s long sample, Figure 5b is the same time-frame sample, but normalized and Figure 5c is a 10 s long spectrogram. The data is raw, unfiltered. The F3–C3 electrode is based on the BANANA montage, similar to the one illustrated with Figure 2a.

Reduction or absence of particular rhythms during the EEG recordings can be considered an abnormality. For example, local brain injury or tumor leads to an abnormal slowing of waves in related areas, spikes, and sharp waves, in turn they may indicate the presence of epileptic foci [26,27,28,32,33,34].

### 2.2. Clinical Applications

The EEG recording is frequently applied to diagnose epilepsy, which causes abnormalities in the EEG readings [33,34]. It can be also used to diagnose sleep disorders, coma, depth of anesthesia, encephalopathies, and brain death [35,36,37]. To the sleep disorders monitored with among the others EEG belong the followings: sleep apnea, insomnia, nocturnal awakenings, excessive sleepiness, narcolepsy, parasomnias, anxiety, restless leg syndrome (RLS), or rapid eye movement (REM) sleep behavior [38,39,40,41,42,43,44].

It is also important to mention that the EEG can be used as a first-line method for diagnosis of tumors, stroke and other brain disorders, but its applicability has decreased with the invention and development of high-resolution anatomical imaging techniques such as magnetic resonance imaging (MRI) and computed tomography (CT) [45,46,47]. Despite the limited spatial resolution, the EEG continues to be a valuable tool for research and diagnostics purposes. It is one of the few mobile techniques available and offers millisecond-range temporal resolution which is not possible to be obtained with the use of CT, PET, or MRI, however, some of the modern systems combine all these methods together—creating the so-called hybrid methods [20,46,47].

The routine EEG is unfortunately often an insufficient source of information to establish appropriate medical diagnosis and to determine the most appropriate treatment, however, some improvements in this area have been made, such as recordings carried out during seizures—ictal recording, as opposed to an inter-ictal recording which refers to the EEG recording between seizures. In some cases—for such purposes—polysomnography can be applied [48,49,50]. The epilepsy recording methods can be also improved with the implementation of various machine learning-based methods, which may help to obtain good quality data [51,52,53].

Epilepsy recordings can be carried out either during hospital admission or under ambulatory conditions (outpatient). The hospital stay is usually performed at the Epilepsy Monitoring Unit (EMU) with appropriate medical personnel trained in taking the appropriate care of patients with seizures. The ambulatory monitoring is usually video-based EEG recording and lasts typically one to three days. An admission to EMU is usually longer and can last a week or longer. While in the hospital, seizure medications are usually withdrawn to increase the odds that seizures occur during the stay at EMU [54]. For reasons of safety, medications are not withdrawn during the EEG recordings performed outside of the hospital [54,55]. The ambulatory video EEG (VEEG) recordings are more convenient (less stressful) for the patients and are less expensive than a hospital admission, but their main disadvantage is that the unsupervised patients are prone to the occurrence of various harmful events and the obtained results depend on the patient’s self-observation ability and can, therefore, be biased [56,57,58,59].

The EEG was not primarily indicated for headache diagnosis, however, some visual evoked potential changes observed in the data may indicate such problem, which may be the reason for headaches occurrence, which are a recurring common pain problem, and this procedure is sometimes used in a search for a diagnosis, but it has no advantage over routine clinical evaluation [60,61,62,63,64].

Besides expensive devices dedicated for clinical measurements there are devices, which are smaller, portable, and cost-effective [20,65,66]. Numerous papers compare various inexpensive EEG headsets, which are mostly applied for the purpose of the above-mentioned BCI systems [5,20,67,68].

Some experiments proved that inexpensive, off-the-shelf devices, despite their lower price, can provide really good data quality (similar to the one obtained from clinical equipment) [20,68,69,70,71,72].

To the most popular inexpensive devices belong those designed and developed by the following companies: Emotiv, OpenBCI, Neurosky or Interaxon [20,67,68]. Their products are becoming more and more popular not only due to their price or recordings’ quality, but also due to their ease of use [67,73,74]. The aspect of ergonomics during EEG measurements is playing a more and more important role [73,74,75].

When it comes to ergonomic or ease of use—some systems require recordings from a smaller number of electrodes to minimize potential data analysis and to ease the montage of the device. Single-channel recordings are becoming more and more popular and some studies proved that these systems still can provide reliable information [76,77,78,79].

### 2.3. Artifacts Present in the EEG Recordings

The EEG signals collected from the surface of the scalp are highly contaminated with various types of internal and external artifacts and background noise [5,80]. It is because the EEG data is prone to disturbances from the inter alia recording unit, amplifiers and cables or motion of other persons around the patient, or by the measured patient itself (as the ECG, breathing, or eye blinking) [5,15,81,82]. Besides the ECG, the EEG recording is influenced more by this noise because of its very small amplitude [82,83]. In the case of insufficient noise removal, the EEG can be totally suppressed and illegible for further analysis and interpretation [16]. The power line interference also frequently occurs in the signal and is one of the main issues, besides EMG- and ECG-related artifacts, in analysis of brain signals (in particular EEG) [84,85]. For such purposes adaptive filters based on a least mean squares (LMS), or those based on normalized LMS [86] can be applied [84,87], or low-pass notch filters [88].

The myopotentials caused by the movements of muscles in the area of the temple and forehead are the most common physiological interference in the EEG signals [89]. However, these potentials take a short time compared to the potentials generated by the brain cells. Moreover, their shape and frequency can easily be identified in the EEG recordings. Some unique patterns of these artifacts can be observed in the case of some movement-related disorders (e.g., rhythmic sinusoidal artifacts of 4–6 Hz for Parkinson’s disease) [90,91].

The low-frequency motion artifacts are usually caused by eye and tongue movements [83,92,92]. The tongue movement results in the wide band of potentials (often within the range of the delta frequency band) which decreases from frontal to occipital area, but it does not reach as expressive amplitudes as the eye artifact [83,92,93,94]. The eye movements are visible in any EEG recording and they are useful for the identification of the sleep stages [95,96]. The motion of the eyeball generates the alternating current with a high amplitude which is visible on electrodes around the eyes [83,92]. Also, the potentials of the muscles of the eye socket are the source of the artifact. Usually, this artifact causes a significant decrease in the EEG amplitude. The slow or steep waves in the EEG signal originate as a result of the breathing synchronously with an inhale or exhale in channels of electrodes, which a patient lies on.

The EEG signal can interfere also with the ECG signal which is easily detectable due to its rhythm and regularity. The voltage level of this artifact differs by measurements. Hence, the ECG signal is ordinarily measured together with the EEG when the effect of the ECG on it can be clearly visible [97,98,99].

### 2.4. EEG Signal Processing Methods

The perturbations induced with various artifacts and random noise are particularly difficult to correct because of their high amplitude, wide spectral distribution, and variable topographical distribution [5,15,16]. Therefore, denoising of the EEG data is a very challenging pre-processing step prior to qualitative or quantitative EEG signal analysis. From the processing methods, both multi-channel and single-channel techniques became widely used, such as e.g., adaptive filters, Wavelet Transform (WT), or Independent Component Analysis (ICA) [100,101,102]. All these methods are summarized in Table 1.

#### 2.4.1. Filtering Methods

For diagnostic purposes—appropriate filtering of the EEG signals plays a crucial role as it can make the data more legible. Therefore, various filtering methods have been tested for many years [103]. Implementation of the traditional, conventional, statistical signal processing methods (such as inter alia: Fourier, Laplace, etc.) can give weak, unsatisfactory results [5,33]. Most commonly applied filters belonging inter alia spatial filters improve significantly signal-to-noise ratio (SNR) [104]. It is hard to choose the most appropriate filtering method due to the non-stationary character of these data and their low amplitude. Most of the typical, classical filters can remove important information from the signal and affect it in a negative way [5,16]. Various smoothing filters, such as inter alia Savitzky–Golay filter can also significantly improve the overall data quality [16]. In many cases, the implementation of the Kalman filters gave very positive results [105,106].

As was mentioned above, more and more channels are utilized in the analysis of the EEG signals, which can impede their potential practical implementation. This one is also one of the reasons for applying appropriate filtering methods [103,107]. In this case (a large number of electrodes applied) spatio-temporal filtering provided satisfactory results [107].

A very interesting area of filtering is non-integer order (fractional) filters. Fractional Calculus is a very interesting mathematical area, which despite being discovered in the 19th century, has become popular very recently [108,109]. They have been successfully applied in numerous different fields such as bioimpedance, image processing, control, encryption, or filtering of biomedical signals [108,110,111,112]. They are useful due to the non-integer order base of the filter, which allows higher flexibility than while using traditional integer-order filters [109].

#### 2.4.2. Wavelet Transform

The WT-based processing methods became popular in biomedical signal processing because of their ability of the high-frequency components suppression and separation of the signal’s low-frequency components [113,114,115], and play a very important role in the analysis and processing of the EEG, mostly of course due to the nature of these signals. Implementation of the Wavelet-filters can be a good solution, in comparison to the traditional signal processing methods, and such a solution was in detail presented in [33]. There are several types of WT such as continuous wavelet transform (CWT), discrete wavelet transform (DWT), stationary wavelet transform (SWT), pitch synchronous wavelet transform (PSWT), etc. The used type of WT depends on input signal and application. Input signal also substantially affects the choice of maternal wavelet and degree of decomposition (for all types of WT). There are a lot of maternal wavelets such as Haar, Daubechies, Biorthogonal, Coiflet, Symlets, Morlet, Mexican Hat, and Meyer.

In [116,117], the authors compare more types of maternal wavelets used for noise removal from the EEG data. The Daubechies wavelets (mainly db8) and Mexican Hat wavelets were considered to be more accurate when recording signals from the healthy subjects (control group) but in the case of the epileptic subjects, the orthogonal Meyer wavelets were more efficient.

#### 2.4.3. Independent Component Analysis

The algorithm of ICA is based on the assumption of the linear combination of the independent sources of signals which are represented with a vector with a mixing matrix which its inverse matrix represents the source of the original signals. The computing of this inverse matrix is the task of the ICA algorithm. In the EEG processing, the ICA is frequently applied for the purpose of EEG signals denoising [118]. The ICA is often used in combination with other filtering techniques [100], such as inter alia Kalman filters [119]. The ICA is also useful in the analysis of multichannel EEG data [120,121].

In [122], the potential of the neural-signal EEG-based methods for enhancing human building interaction under various indoor temperatures were explored. As a pre-processing, the Matlab toolbox, which contains the HPF with a cut-off frequency 3 Hz to remove the DC offset and the low-frequency skin potentials artifacts, and then the LPF at 45 Hz to remove high-frequency noises, was used. Non-stereotyped artifacts such as large movements noises were visually rejected by manually scrolling the data and the remaining artifacts, such as eye blinks and muscle activities were removed with a built-in ICA algorithm.

Corradino et al. [123] used the ICA-based methods for the detection of artifacts in the EEG which were removed with another algorithm, the ordinary least square. The matrix of independent components was further filtered with a notch filter because there was a presumption of a certain frequency of the detected artifact. The procedure proved to be effective but not suitable for online signal processing due to the implementation of the time-consuming ICA algorithm.

Li et al. [124] proposed the method which includes the following three steps: independent components decomposition, the common interference identification, and its removal. For the ICA implementation, FastICA was adopted with the maximum-negative entropy principle. The putative common interference component is assumed as a distal signal, which had approximately the same effect on all channels. To obtain local brain activities more accurately, these distal common interference components should be removed.

Arnin et al. [125] presented the signal processing method for the purpose of BCI development which was introduced for severe neurological diseases especially for the locked-in patients, who are severely or totally neuro-muscularly impaired, which means, they are unable to move or speak, so their only way for communication is the use of brain signals [126,127]. The concept is to detect one’s movement intention and use it to control external devices such as wheelchair or rehabilitation equipment. After exploring the EEG data-set, the present artifacts were reduced using the ICA and the result was an artifact-free signal, which was later processed using different feature extraction methods [128,129,130].

As it has been stated above—the ICA is an efficient and popular method in the analysis of various biomedical data, in particular EEG [120,121]. It is a type of BSS (Blind Source Separation) method usually applied in the analysis of the EEG signals to separate various artifacts, such as eye blinks or muscle artifacts [120].

Most of the ICA-based algorithms require large amounts of training data and are suitable for offline solutions only assuming spatio-temporal stationarity of the analyzed data, as in popular e.g., Infomax ICA and FastICA, which are also computationally expensive and time-inefficient [120,121].

In [120], the authors presented ICA-based algorithm on large 61 channel data for the purpose of online brain and artifacts decomposition and obtained interesting results, proving its efficiency in online solution. They applied an algorithm called ORICA (Online Recursive Independent Component Analysis), which was compared with the offline Infomax. The results were very promising and proved that the ORICA is a very powerful tool for online EEG data analysis [120,131].

#### 2.4.4. Empirical Mode Decomposition

Empirical Mode Decomposition (EMD) method is often used for biomedical signal processing due to its adaptability and signal-dependency. The algorithm extracts the individual Intrinsic Mode Functions (IMFs) in each iteration from detected maximum and minimum extremes. Such decomposition is advantageous in eliminating the low-frequency noise and narrow-band information [132]. The method allows processing single-channel signals in contrast to ICA.

Amo et al. [133] determined whether detection of gamma-band activity can be improved when a filter, based on the EMD, was added to the pre-processing block of the single-channel EEG signals. In their study—the EEGs from 25 control subjects were registered in basal and motor activity (hand movements) using only one EEG channel. Over the basic signal, the IMF signals were computed. The gamma-band activity was computed using power spectrum density in the 30–60 Hz range.

Chen et al. [134] used the EEG signals obtained from patients in two different states—an aware state and anesthetized. The data was decomposed into a set of the Intrinsic Mode Functions (IMFs) with the implementation of the EMD algorithm. The Fast Fourier transform (FFT) and Hilbert transform (HT) analyses were then performed on each IMF to determine the frequency spectra. The probability distributions of the expected frequency values were generated for the same IMF in the two above-mentioned groups of patients. The corresponding statistical data, including analysis of variance tests, was also calculated. A receiver operating characteristic curve was used to identify the optimal frequency value to discriminate between the two states of consciousness.

Gaur et al. [135] used a BCI system to translate human motion intentions using signals generated with the electrical activity of the brain such as the EEG into control signals. One of the major challenges in the BCI research is a classification of the human motion intentions from the non-stationary EEG signals [136]. In [135] a novel subject-specific multivariate EMD-based filtering method was proposed, namely, the SS-MEMDBF (subject-specific multivariate empirical mode decomposition), which classifies the motor imagery (MI) based EEG signals into multiple classes. The MEMD method simultaneously decomposed the multi-channel EEG signals into a group of multivariate IMFs (MIMFs). This decomposition enabled the extraction of the cross-channel information and also localization of the specific frequency information. The MIMFs are considered as narrow-band, amplitude, and frequency modulated signals. The EMD-based method can be also applied for the purpose of muscular interference removal and to clean the signal from the EOG artifacts [137].

#### 2.4.5. Time-Frequency Image Dimensionality Reduction

Most of the EEG processing methods, such as the ICA, are only available for the signal processing of the multi-electrode EEG. However, the results obtained from only single channels are becoming more and more popular [77,89,138]. For the single-channel EEG, methods such as WT or adaptive filters are very popular, but they just suppress the noise outside the filtering frequency band, while the co-channel interference is left unprocessed [138]. For the co-channel interference removal, a method based on the time-frequency (T-F) image dimensionality reduction was proposed in [97]. The innovative idea of the proposed method was that it can be applicable for a single electrode EEG signal enhancement and the background noise can be suppressed in the entire time-frequency space. The proposed method was experimentally validated with a set of real EEG data. The experimental results indicated that the proposed method was effective in the EEG single electrode co-channel interference suppression.

#### 2.4.6. Neural Networks

Denoising of EEG based on neural networks started to be investigated mainly due to its usability in real-time and, of course, high accuracy. For example, Leite et al. [139] proposed a deep convolutional auto-encoder for eye-blinking and jaw-clenching artifacts elimination, which proved the superiority to traditional filtering methods. The elimination of ocular artifacts (OAs) using a deep learning network (DLN) was investigated in [140]. The filtration was performed in two stages; in the offline stage, training samples without artifacts were used to train DLN to reconstruct the EEG signals. After, this trained DLN is used as a filter to automatically remove OAs from the contaminated EEG signals in the online stage. The performance of the proposed method was compared with the classic ICA, kurtosis-ICA, second-order blind identification, and shallow network method. Besides the higher success rate of eliminating artifacts, the method brought other advantages, such as no-needed additional EOG reference signals, the possibility of analyzing a smaller number of channels, and time savings.

In another study [141], ocular, muscular, and cardiac artifacts were removed from EEG using an adaptive filter based on radial basis function network and functional link neural network (called FLN–RBFN-based filter). This method was tested in both offline and online modes. While offline learning algorithm has simpler computations and assumes the EEG signal stationary, a more demanding online algorithm is updated based on the new incoming patterns in each cycle, thus well meets the non-stationarity of the EEG signal. However, both approaches work well in artifact removal with significantly considerable improvement in results. Real-time EEG denoising was also proposed by Cowan et al. [142] by using the smart electrode employing DNN that learns continuously to remove both eye-blink and muscle artifacts and works based on adaptive filtering.

#### 2.4.7. Adaptive Neuro-Fuzzy Inference System

Adaptive Neuro-Fuzzy Inference System (ANFIS) is an adaptive soft-computing method based on analytical methods, Boolean logic, sharp classification, and deterministic searching. The soft computing methods are those, which include among the others: artificial neural networks (ANN), genetic algorithms (GAs), fuzzy logic (FL), adaptive neuro-fuzzy inference systems (ANFIS), support vector machines (SVM), and data mining (DM) [143]. The ANFIS combines fuzzy inference function and artificial neural network, taking advantage of both powerful techniques. The basic idea of ANFIS is an architecture utilizing a fuzzy system to represent interpretable knowledge and the learning capability of neural networks to optimize their parameters [144].

Adaptive filtering of OAs using ANFIS was proposed by Chen et al. [145] on both simulated and real signals. In the first case, the ANFIS outperformed adaptive filtering and ADALINE (Adaptive Linear Element) methods. The authors also investigated the influence of time delay of OAs, which occurs in real EEG measurement. Here, it was found that the number of ANFIS inputs plays a role in the efficiency of the artifact removal—using a higher number resulted in slight improvement at the cost of a longer convergence time. This sensitivity of the chosen number of inputs was also proved in real data sets. In this case, the ANFIS was also considered a suitable tool for OAs and other noise removal.

The elimination of OAs by ANFIS was also presented in [146], where the method was improved using a genetic algorithm to optimize the parameters of the ANFIS structure. The proposed method reached better performance in SNR compared to the traditional ANFIS algorithm, especially when a higher number of iterations was used.

#### 2.4.8. Hybrid Methods

A combination of at least two individual techniques is considered a hybrid method in this paper. These methods are beneficial due to their performance and accuracy. However, their implementation has higher requirements on computational cost and complexity in contrast with other above-discussed methods and thus, their application has to be considered depending on its purposes.

To improve pre-processing of EEG data, a combination of WT and ICA was proposed in [147,148]. The principle of this method lies in the separation of artifact-independent components, filtering of this component by WT to remove any brain activity, and finally, project back the artifacts to be subtracted from EEG signals to get clean EEG data. The method proved its efficiency in artifact removal and computational time with respect to particular ICA approaches.

For the removal of OAs, the combination of the WT-based and the ANC-based methods was useful [149,150,151]. The reference input was derived from the contaminated EEG, it was uncorrelated with the EEG but had a strong correlation with the OAs and met the conditions of the ANC as a reference input. After the wavelet decomposition (using the appropriate maternal wavelet and decomposition level), soft thresholding was applied on the three lowest levels. The reference signal was obtained from the application of a wavelet reconstruction to these new wavelet coefficients. The OAs can be eliminated with the implementation of the ANC filter with the reconstructed reference signal [152,153].

Jafarifarmand et al. [154] investigated real-time processing and OAs removal using a combination of ICA and ANC. In this approach, ICA is applied on only a few EEG channels close to the artifact origin. The resulting independent component, most relevant to the artifact, is then used as a reference signal for ANC, employing fully automated neural networks. Due to real-time operation and a small number of channels required, the method is promising for BCI applications.

Torse et al. [155] compared the several EEG pre-processing methods such as FastICA, runICA, the Principal Component Analysis (PCA), and adaptive filtering, both with and without a reference signal, examining their effect on the EOG artifacts and epileptic recording. The methods were compared in accordance with the two aspects: middle quadratic errors and computational times. Adaptive filtering differs significantly from the other popular methods by its short computation time, but at the expense of accuracy, which is achieved much higher than the other methods; ICA (most accurate) and PCA (fastest but lower accuracy than ICA). The application of ICA provided very good results in removing eye-blinking artifacts [101,156].

### 2.5. Summary of the EEG Signals’ Processing Methods

Table 1 summarizes the recently used EEG signal processing methods. The following criteria were chosen for the evaluation of their performance and comparison (same criteria is used in Table 2 and Table 3):

- **Overall performance** combines other used criteria (SNR improvement, computational cost, real-time and implementation complexity) and gives overall evaluation which reflects the robustness of the method in three categories:
  - *Low*: enables to remove some specific types of interference but the original signal is quite distorted.
  - *Medium*: the signal can be preserved when using the proper parameters for noise removal, which are difficult to choose.
  - *High*: the signal is processed with a preservation of its original shape, so the detailed evaluation of all signal parameters is possible.
- **SNR improvement** classifies the efficiency of the method with regards to the reference in three categories:
  - *Low*: these methods are suitable primarily for signal preprocessing (reducing baseline wandering, power line interference, etc.) and improvement is ≤5 dB (based on experiments with synthetic records).
  - *Medium*: these methods are suitable primarily for signal preprocessing (power-line interference, myopotentials, and electromyographic interference, isoelectric line fluctuations, motion artifacts, etc.) and improvement is ≤20 dB (based on experiments with synthetic records).
  - *High*: these methods are the most powerful comprehensive processing methods that provide very important information that other methods do not allow. Improvement is ≥20 dB (based on experiments with synthetic records).
- **Computational cost** determines the demands of the methods in terms of computational complexity in three categories:
  - *Low*: suitable for use in real-time applications. Provide a good compromise between computational cost on the device’s memory and carries out the calculation faster.
  - *Medium*: real-time application is possible but only with advanced technology, such as powerful computers or circuits with field-programmable gate array (FPGA).
  - *High*: the design is too complex and thus not suitable for real-time and/or low-cost applications.
- **Real-time** is a parameter defining whether the method can be used in online mode, which is very desirable for usability in clinical practice.
  - *Yes*: these methods are suitable for real-time applications.
  - *No*: these methods are not suitable for real-time applications or applications where a small delay is critical.
- **Implementation complexity** classifies the overall complexity in terms of the deployment in clinical practice to evaluate the economic availability of hardware and software to all patients.
  - *Simple*: these methods are composed of well-known functions and basic mathematical operations, so it is simple to implement them
  - *Medium*: these methods contain advanced signal processing algorithms that are not commonly available and thus harder to implement.
  - *Complex*: these methods contain advanced signal processing methods and complex algorithms making it very challenging to design and implement them.

The presented comparison is highly subjective and based on the earlier experience of the team of authors. Our results were verified by a set of basic experiments on a predefined signal sample.

### 2.6. Other Methods—Brief Summary

As it has been mentioned above, in BCI system, many methods applied for feature extraction have been proposed and applied, such as among the others: analysis of raw time-series, signal power estimation, etc. [20,157]. Most of these include classical, typical methods such as various Fourier transforms or wavelet decomposition, which belong to the classic methods applied for signal processing purposes [5,157]. One of the most interesting, although, not explored significantly is a cross-frequency coupling (CFC) method, which has a wide applicability potential for the BCI systems [157,158]. The CFC defines different phenomena related to interactions between different frequency oscillations in the human brain [159].

One of the very interesting studies involving CFC was presented in [157], where the authors focused on decoding brain signals generated by the flashing images using the CFC estimator—PAC (phase-to-amplitude coupling) to see how the phase of the lower frequency ranges affects the higher oscillations. Their study proved the CFC to be an efficient and valuable estimator. Similar results regarding CFC-PAC were also shown in [160,161].

The use of CFC is being applied usually for cognitive and perceptual processes purposes. Despite some promising results [157,160,161] it has some limitations, as it is flexible only either over time or over frequency [162]. For example, Michael Cohen et al. presented a method for transient cross-frequency assessment in [158,162], which enables multiple, dynamic, and CFC structures over time and frequency.

In [163], various CFC measures were tested on both real and simulated EEG signals. The obtained results were as follows: the CFC detection was correct under noisy conditions; the CFC detection was correct in simulated data; prominent delta-alpha CFC was identified in the real, resting-state EEG.

It is also important to mention functional brain connectivity, which can be defined as the temporal correlation among the activity of various neural assemblies. With the use of functional connectivity techniques the following brain signals (data) can be derived: local field potential (LFP) recordings, Electroencephalography (EEG), Magnetoencephalography (MEG), Positron Emission Tomography (PET), and Functional Magnetic Resonance Imaging (fMRI) [164].

Interesting approach in various methods assessment based on simulated EEG signals was presented in [165]. The authors of the work decided to focus on highlighting potential pitfalls while using various numerical methods instead of proving information regarding their efficiency. On the other hand, in [164], various mathematical methods for functional and effective connectivity calculation in both EEG and MEG signals were presented, in particular endeavor, linear, and non-linear.

For various diseases diagnostics purposes, such as Epilepsy [166,167], Parkinson’s disease [168] deep learning-based methods are being frequently applied [169]. In order to improve the diagnostics processes the use of automatic systems, based on neural networks (e.g., CNN) or various expert systems are being developed and applied [166,167,170].

In [166] the obtained accuracy regarding Epilepsy detection was 99.1±0.9, which is an excellent result. The authors applied Convolutional Neural Networks. In [168] the authors presented an interesting automated system of Parkinson’s detection, based in CNN architecture, with 88.25% accuracy, 84.71% sensitivity, and 91.77% specificity. The obtained results are very good and promising.

Besides medical purposes, deep learning techniques in the analysis of EEG data can be also applied for biometrics purposes [171] or real-time IoT (Internet of Things) applications [172], or for emotions’ recognition purposes [173].

## 3. Evoked Potentials

As a derivative of the EEG recording—evoked potentials (EPs) or event-related potentials (ERPs) can be observed as a brain’s reaction to external stimuli (visual, somatosensory, or auditory) [174,175]. The EPs refer to the averaged EEG responses, which are time-locked to more complex processing of stimuli; this technique is used in cognitive science, cognitive psychology, and psycho-physiological research, they can also be applied as biometric indicators [175]. The EPs are weak signals, characterized by a very low amplitude in comparison to the full EEG data, and are often buried in the activity of associated systems, because the response to the external stimuli is generated with only a small percentage of the brain neurons. Therefore, their SNR can be improved using the synchronized averaging or filtering, and the repeating of the stimuli ten to thousand times. The repetition of the same stimuli causes the response with similar characteristics. The objects of interest are the signal’s amplitude, length, and the repeating of the stimuli ten to thousand times. The repetition of the same stimuli causes the response with similar characteristics. The objects of interest are the signal’s amplitude, length, and latency of the response on the stimulus [174,176].

The common, classic surface EEG electrodes are usually used for the recording of the EPs, except for intra-operative sensing from the cortex, where the cortical strips or grids are placed. The placement of the electrodes is the same as in the EEG measurements (“10–20” system, see Figure 1). The leads are different from the classical EEG system and they are not internationally standardized. It is possible to use both unipolar and bipolar leads, usually a maximum of four channels [26,27,176].

### 3.1. EP Recordings

**Figure 6:**
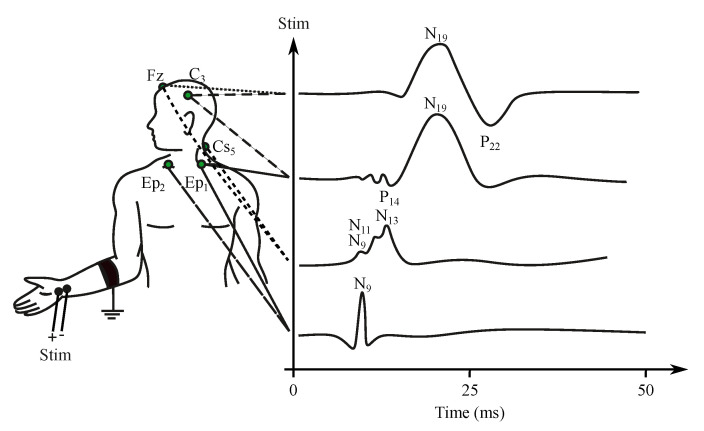
Example of the SEP signal.

**Figure 7:**
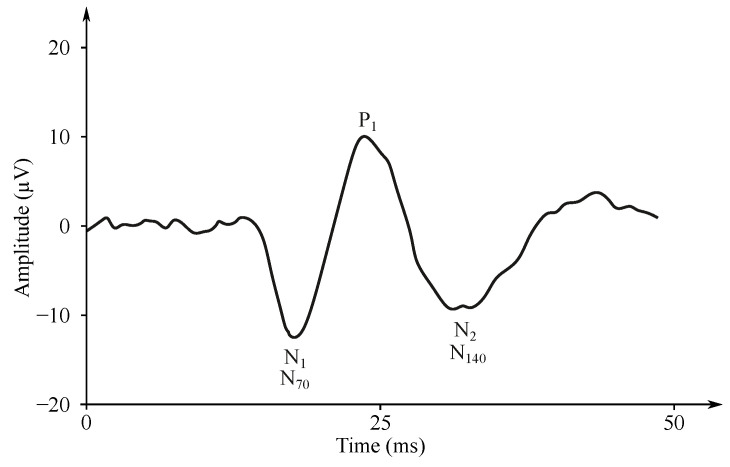
Example of AEP signal [177].

The characteristic of the EP (event potentials) recording depends on the type of the applied stimuli. The response can be ipsilateral when the hemisphere corresponding to the half of the body where the receptors were stimulated reacts on the auditory EPs. On the contrary, the contralateral response can be evoked with visual and somatosensory stimuli, when the opposite hemisphere is activated [26,27]. A nomenclature of the EP waveform can be derived from the two following methods:

- *Somatosensory evoked potentials (SEP)*—are generated by the nervous system following a somatosensory stimulus. For this method, the components are labeled according to the polarity and mean latency in normal subjects, e.g., P100, N20 (see Figure 6) [176].
- *Auditory evoked potentials (AEP)*— follow audio stimulation. For this method, components are numbered according to their polarity in sequence, e.g., N1, N2, N3 (see Figure 7).

#### 3.1.1. Somatosensory EP

The somatosensory EP (SEP) represents the response to the stimulation of the receptor fibers of the peripheral nerves, most often of the limbs. Stimuli are generated with electrical excitation using a neuro-stimulator and surface electrodes placed close to the nerve (e.g., nervus medianus in the area of the palm, nervus peroneus in the area of knee-joint) [178,179]. The stimulating impulse has a length from 50 μs to 1 ms and is repeated 200 times with a frequency of 3–10 Hz. The current of the stimuli gains the amplitude of about 25–50 mA [180,181].

The length of the recorded response is about 40 ms in the case of the upper limb and 60–80 ms in the case of the lower limb. The SEP signal has a frequency range of 30–3000 Hz. The voltage of the signal is very low, typically only 5–10 μV. After the stimulation, the certain time interval, which reflects the time necessary for excitation transmission from the peripheral nerve to the somatosensory cortex, is held before the signal recording (25 ms for upper limb, 40 ms for lower limb). This time delay is often the main indication of pathological characteristics [26,27,182].

#### 3.1.2. Auditory EP

The auditory EP (AEP) is investigated with short-time sounds (100 μs) with different frequencies, so-called clicks using headphones in only one ear or both ears at the same time. The AEP stimuli are repeated 2000 times with the frequency of 1–50 Hz [183,184,185].

The signal is measured with the electrodes placed on the processus mastoideus on the temporal bone just behind the auricles. The amplitude of the recorded signal reaches about 0.5 μV [27]. Waveforms of the AEP can be represented in three levels of latency, i.e., early, middle and late response, which are distinguished by the different denominations, see Figure 6 [181,186,187]. The correlation between the click and the obtained EEG signal determines the sensitivity of the central nervous system to the played sound, which can be diagnosed as pathological when the recorded signal does not occur on the specific frequency [182]. The AEP can be applied for the purpose of brain death estimation or to check the level of anesthetics [186,188].

#### 3.1.3. Visual EP

**Figure 8:**
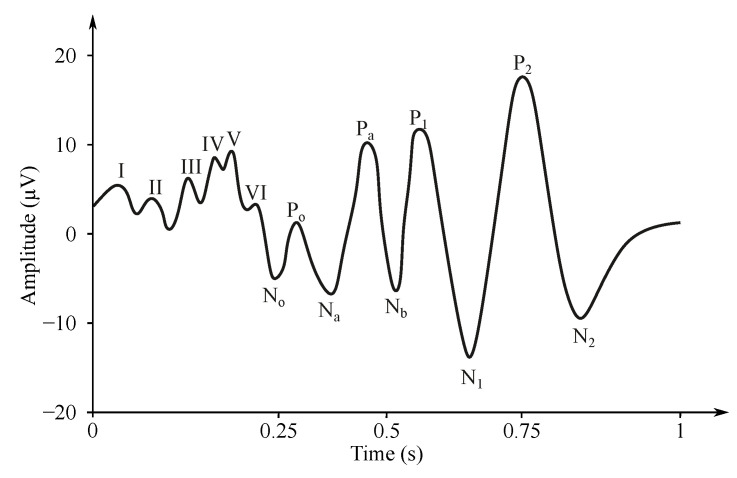
Example of the VEP signal [192].

For the examination of the visual EP (VEP) (see Figure 8), usually chessboard reverse stimuli are used [189,190]. The subject observes the computer monitor (or special glasses) with the chessboard containing black and white boxes [189,190,191]. The boxes swap their color in the periodic intervals (white boxes change to black and vice versa) with a frequency of 1 Hz. The VEP stimuli can be repeated only 100 times because a large number of neurons is involved in the reaction, and the activity of the visual analyzer is well localizable [189].

The sensing takes place on the occipital cerebral lobe, where the visual analyzer is located. The frequency range of the signal is narrower than the other modalities, up to 100 Hz. The signal amplitude gains a voltage of 5–10 μV. A reaction to the stimulus comes after 100 ms [27,182].

#### 3.1.4. Event-Related Potentials (ERPs)

The ERPs represent a brain examination very closely related to the EPs examination. They investigate the response of the central nervous system to psycho-physiological events when detecting the EEG resulting from a combination of various stimuli [193,194,195]. The stimuli emulate a certain environment, for example, smell, electric currents, and muscle stimuli [194]. The most common stimuli for the ERPs are visual patterns and light, similarly to the VEPs, but the difference is in the combination of the basic stimuli usually used in the EPs, so that the brain is tested for its response to complicated tasks, when arranging the stimuli according to the specific archetypes [195].

The ERP signal is very small in amplitude, so commonly, the repetitive measurements are performed and signals are averaged, which would increase the SNR [182].

### 3.2. Clinical Applications

The SEP is used in diagnostics of neuropathy, multiple sclerosis, and other pathological conditions caused by nerve demyelination. This examination can also be applied for evaluation of the depth of coma or anesthesia and to prognosis assessment [177,186,188].

The AEP enables the determination of various disorders such as cochlea or acoustic nerve, which are expressed with the lower amplitude of the response. The examination enables detection of both mechanical and neurological hearing damages. The delayed responses may point at lesions of the brain stem. Certain pathological auditory conditions can be treated with the implementation of various surgical methods [196].

The VEP examination is applied in the diagnostics of multiple sclerosis when the demyelination of the optic nerve occurs and can be used also for the determination of the difference between right and left vision [197,198,199].

The measurements of the ERP are often used to investigate the neurophysiologic correlation between factual knowledge, awareness, and attention, and they can also be used to identify specific components or patterns in the myopotentials [26,27,182].

### 3.3. EP Processing Methods

Because the EP is a signal measured in the same way as the EEG signal, the similar artifacts and interference can affect them, such as background noise, movement of patient or electrodes, and other electrical activity of the patient’s body (cardiac and ocular functions, breathing or myopotentials) [200,201], what was described in more detail in Section 2. In addition, the EP signal is usually contaminated with the spontaneous EEG signal of the brain, which gains multiple amplitudes. When measuring the SEPs, the stimulus artifact is present and possible to be observed in the signal, as a reaction on the electrical impulse stimulating peripheral nerves. A similar problem arises when stimulating a cochlear implant in the AEPs [94,202,203].

Many publications dealing with the problems of the EP signals measurement focus on the stimulus artifacts removal [204,205,206]. The elimination of this artifact is more challenging than the simple filtering of the analyzed signal, because the frequency spectra of the EP signal and the applied stimulus often overlap, so the filtering of the stimulus frequencies can distort the desired EPs. The so-called sample-and-hold method (see [207,208,209,210,211,212,213]) has been the most widespread method for over 20 years when the circuit switches to the “hold” mode immediately prior to the used stimulation so that the recording amplifier is prevented from the stimulus artifact detection. After that, the circuit moves back to the “sample” mode and passes the signal to the amplifier. This technique is useful in case of the clear separation of the artifact from the signal in time. However, the estimation of the “hold” mode duration plays a significant role, because when it is too long, the desired signal can be completely removed from the recording, and if too short the residual artifact remains in the signal affecting it in a negative way. Thus, the sample-and-hold method was suitable only for studies using low-rate electrical stimulation and investigating long latency responses, so for other types of examinations, the more sophisticated methods should be used. Thus, with the conversion of the signal from the analog to the digital form and the associated development of modern software techniques, many signal processing methods were deeply investigated.

In the case of using a high rate of stimuli, which is 200–5000 pulses/s—Heffer et al. [214] used a sample-and-interpolation technique to remove the stimulus artifact events with minimal distortion of the desired action potentials [214,215]. As a pre-processing step an HP filter was applied to remove the DC offset and the baseline drift under 5 Hz. This method was based on a prior knowledge or previously conducted measurement of the maximum stimulus duration from the recorded data, followed by the identification of stimulus artifact events with a computer control stimulation or using amplitude threshold level crossing, where the threshold is a minimal value of the stimulus current needed to induce a neural response. The proper removal of the occurring artifacts was then realized with the determination of a sample point prior to the stimulus artifact and a sample point following the end of this artifact, the interpolation straight line between these two points and replacing the original samples with the interpolated values. The method successfully eliminated the high-rate stimuli with the amplitude several times higher than the EP signal, when the signal was practically lost in the interference.

The other area of interest in the processing of the EPs is the extraction of the desired signal from the background brain activity (the spontaneous EEG), which enables to obtain single-trial response instead of averaging a large number of responses [216,217]. Because the frequency ranges of the EPs and the background EEG overlap, it is more advised to implement sophisticated methods than the classic filtering. As an example of a sophisticated method the Wiener filter or the adaptive algorithms can be considered (see [218,219,220,221,222,223]). However, with the development of computing technology, other methods could overcome these techniques, such as the following: WT, ICA, or Principal Component Analysis (PCA) [224]. The summary of these techniques, their properties, and their efficiency are listed in Table 2.

#### 3.3.1. Wavelet Transform

Quiroga et al. [225,226] dealt with the denoising of a single trial of the EP recording to increase the diagnostic information, which can lie in the variations in responses to stimuli. In this study, the extraction of the single-trial VEPs and AEPs from the background EEG was made with the implementation of the WT method using quadratic biorthogonal B-splines as the mother functions. The filtering procedure was based on the wavelet decomposition of the single-trial at five levels, and further the identification of the components non-correlated with the average EPs signal when setting them to zero. The inverse transform then brought the denoised single-trial response. par In another study, Quiroga and Garcia [227] proposed the WT denoising based on the decomposition of the average ERPs signal, when the coefficients correlated with the ERPs signal were identified and those remaining were simply set to zero. The single trial was denoised using the obtained coefficients and then reconstructed. This procedure gained the lower RMSE values in comparison with the previously mentioned Wiener filter, which is also frequently used for the EPs signal’s denoising.

Ahmadi et al. [228] proposed an automatic denoising implementation for visualization of a single trial of the ERPs based on the WT with an automatic selection of wavelet coefficients based on the inter- and intra-scale correlation of the neighboring wavelet coefficients and their deviation from the signal’s baseline. For the denoising of the single-trial ERPs, the coefficients were obtained with these followings: (1) the wavelet decomposition of the average ERPs, and (2) the hard thresholding and the so-called zero-trees procedure, when the not significant wavelet coefficients with respect to the given threshold were deleted (see [229]). The method shows significant improvement in the process of identification of the amplitude and the latency of the evoked responses, and the lower RMSE in comparison with the original signal and the other WT procedures based on thresholding.

Wang et al. [230] proposed the WT denoising of the EPs using the Daubechies family of wavelets and soft thresholding. The results were compared with the filtering using Wiener filter and the adaptive algorithms (RLS, LMS) when the reference signal was calculated as the ensemble average of all trials except the trial, which is being considered. The WT-based approach reached the SNR values significantly higher and the RMSE values significantly lower than the other filtering methods. The WT method was found to be more effective for the EPs extraction.

#### 3.3.2. Independent Component Analysis

Iyer et al. [231] dealt with the denoising of the AEPs with the implementation of the iterative ICA. The ICA approach is based on the idea that the activity resulting from an experimental stimulus is independent of the neuro-physiological artifacts and the background EEG. The advantages of the ICA in the area of the EPs denoising are the extraction of the individual components and the estimation of these components in the single-trial signals. The process of denoising the single-trial includes the correlation computation between the average EP and the independent components obtained with the application of the ICA on all the blocks of 10 single trials. The independent components, which are correlated less than a defined threshold, are set to zero. According to this study, the iterative ICA technique provided better results than the WT in the estimation of single-trial responses and elimination of the background brain activity, thus distinguishing the important components of the EP.

Zouridakis et al. [232] also proposed the ICA-based denoising of the EPs in comparison with the implementation of the WT. The iterative process allowed the components of interest to be distinguished better—peaks become sharper and conversely, the data outside the region of interest became flatter. Also, peaks occurred at nearly consistent latency with some jitter. When using filtering with the WT there is a wide variation in the latency of the interest components.

Lee et al. [233] presented the ICA method for the processing of the VEP signals. The filtered EEG data (in the range of 0.1–50 Hz) was decomposed into independent components with the FastICA algorithm, and the correlation between spatial maps of independent components and predetermined spatial template was calculated to select the components for data reconstruction. The method was efficient for the elimination of the ongoing EEG signal and related artifacts.

In [234] was presented a solution for EP analysis, where the Independent Component Analysis (ICA) was combined with fuzzy clustering, which can be an interesting way for further investigations.

#### 3.3.3. Principal Component Analysis

Principal Component Analysis (PCA) is a very popular signal processing method and is based on a similar principle as the ICA but here the signal is decomposed to linearly uncorrelated components (not statistically independent, as in case of the ICA).

Palaniappan et al. [235] proposed denoising of the VEP signal with the application of the PCA technique, which would be useful to retain the most important components from the signal, and to denote both noise and the background EEG. After computing the principal components, those with eigenvalues greater than 1.0 were considered to be part of the VEP subspace, while the rest were marked as noise. The reconstructed signal was then filtered with the Butterworth pass-band filter of 30–50 Hz, and the resulting data were normalized. The authors investigated the effect of this processing step on the outcome of the VEP feature extraction, and claimed that both the PCA and the normalization improved the classification performance up to 96.50% (the lowest improvement was 66.12%). Also, the PCA processing helped to reduce computational complexity and time algorithms classification.

The PCA method with two levels was proposed in [236]. The first level included decomposition of the matrix of the VEP channels. This step removed noise from the signal, which was random in contrast to the highly correlated brain signal in individual channels. The principal components with eigenvalues higher than 1.0 were again used for the reconstruction. The resulting denoised signal was then decomposed, as the second PCA level, and was applied across single trials (not across channels of a trial as previously) to remove the background EEG signal.

Mowla et al. [237] proposed the iterative PCA algorithm for the EP estimation. The PCA was applied to each group of 10 randomly assigned single trials. The correlation between the principal components and the average EP was computed and if its value lied below the defined threshold, this component was considered to contain mainly the background EEG. Thus, this principal component was replaced with zeros to eliminate the noise. The percentage of correct latency for the iterative PCA reached 97%, while using other methods (the iterative ICA and canonical correlation analysis), it was around 70%.

#### 3.3.4. Hybrid Methods

Hu et al. [238] proposed a method for laser processing of the EP signals using a combination of the ICA and the WT with the main aim to reach the higher SNR. As a first step, the recorded signal was filtered with a Band-Pass (BP) filter between 1 and 30 Hz, and the artifacts caused by the eye movements and blinking were removed using the ICA. After that, the low-SNR components were detected again using ICA and their SNR was improved by using WT.

Zou et al. [239] proposed a new approach for the reduction of the number of required trials for efficient extraction of the ERPs by combining the WT and the PCA methods. First, the SNR of the ERPs was improved using the WT, so the principal components could be extracted. Then, the selected principal components were used to reconstruct a denoised signal. The obtained results showed higher values of the SNR and the lower RMSE than each method used separately.

## 4. Electrocorticography

**Figure 9:**
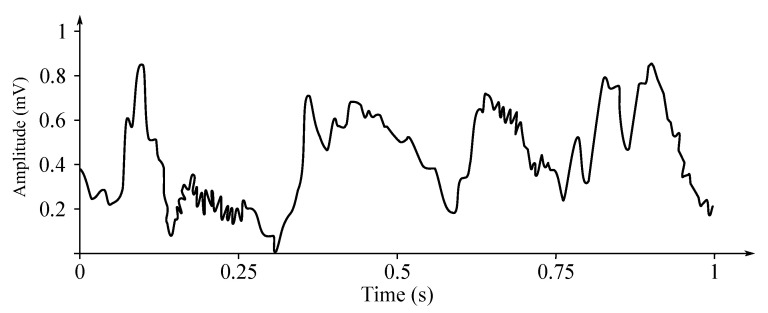
Example of the ECoG signal.

If a patient with epilepsy is being considered for a resection surgery, it is often necessary to localize the focus (source) of the epileptic brain activity with a resolution greater than what is provided with a classic scalp-based, surface EEG [240]. This is because the cerebrospinal fluid, skull, and scalp confound the electrical potentials recorded with the background EEG. In such cases, the neurosurgeons typically implant strips and grids of electrodes (or deep electrodes, which would penetrate the study area) under the dura mater, through either a craniotomy or a burr hole. The recording of these signals is referred to as electrocorticography (ECoG), subdural EEG (sdEEG), or intracranial EEG (icEEG) [5]. The signal recorded from the ECoG has a higher voltage than the data recorded from the surface EEG [240]. Also the low voltage, high-frequency components, which cannot be seen in signals recorded with the classic scalp EEG can be seen clearly in the ECoG recordings. Furthermore, smaller electrodes, which cover a smaller parcel of the brain surface, enable recording of even lower voltage, faster components of the brain activity. Some clinical sites record from the penetrating micro-electrodes, used in case a tissue with the implanted electrode is damaged, so such penetrating electrode “moves” and in result allows recording from areas with healthy tissue [5]. The ECoG is performed mainly on patients who will have to have a follow-up resection procedure for sure [241,242]. Figure 9 shows an example of the ECoG signal.

### 4.1. ECoG Recordings

As the ECoG signals consist of synchronized postsynaptic potentials (local field potentials) recorded directly from the exposed crust surface, which occur mainly in the cortical pyramidal cells. These must overcome several layers of the cortex, cerebrospinal fluid (CSF), pia mater, and arachnoid mater before reaching the subdural recording electrodes located just below the dura mater. In order to reach the electrodes placed directly on the scalp (conventional electroencephalogram—EEG) the electrical signals must also be conducted through the skull, where the potentials decrease rapidly due to the low conductivity of the bone. The lack of such “obstacles” makes the spatial resolution of the ECoG much higher than the one obtained from the surface EEG, which is a critical imaging advantage. The ECoG offers a time resolution of approximately 5 ms and a spatial resolution of 1 cm [5,20,240,243,244].

The local field potential obtained from the depth electrode provides a measure of the neural population in a sphere with a radius of 0.5–3 mm around the tip of the electrode. At a sufficiently high sampling frequency (more than 10 kHz) they enable to measure the action potentials. In this case, the spatial resolution of the individual neurons and the field of view of the individual electrode oscillates around 0.05–0.35 mm [5,240,245,246].

### 4.2. Clinical Applications

The ECoG is used mainly to locate the epileptogenic zones during preliminary planning, mapping cortical functions, and prediction of the success of epileptic surgical resection. The ECoG, despite its invasiveness, has several advantages over alternative—non-invasive diagnostic methods [242]:

- Flexible placement of recording and stimulation electrodes;
- It can be performed at any stage before, during and after surgery;
- It allows direct electrical stimulation of the brain and identification of critical areas of the cortex, which must be avoided during surgery;
- It provides greater accuracy and sensitivity than the scalp EEG recordings, as the spatial resolution is higher and the signal-to-noise ratio is better due to closer proximity to the neural activity.

Limitations of the ECoG [247]:

- Limited sampling times recording may be impossible;
- Electrodes’ placement is limited with the area of the exposed cortex and the time of surgery, which causes limited view field and sampling errors’ occurrence;
- The recording is influenced by the anesthetics, analgesics, and the surgery itself.

### 4.3. ECoG Processing Methods

Because the ECoG is a signal measured from the electrical activity of the brain (similar to the EEG), although it requires surgical intervention and therefore is considered to be invasive, it is also very prone to the occurrence of similar internal and external artifacts to those affecting classic EEG recordings, such as inter alia: background noise, patient movement (the ECoG is performed under general anesthesia, so this artifact is a minor consequence) or electrodes and other electrical activity of the patient’s body (cardiac and ocular functions, respiration or myopotentials) [248,249].

The electrical activity recorded from the cortical surface of the brain represents the average of many individual synaptic processes. With reduction of the micro-electrode fields, the spatial resolution of the electrocorticography (ECoG) can be increased. However, as the electrode impedance increases, more significant noise is recorded from the electrode-tissue interface and the environment [247]. Signal interpretation is often improved with post-processing through filtering or application of various pattern recognition methods [248,250].

Spatial spectral analysis is necessary to derive spatial patterns from the current ECoG recordings to determine the optimal interval between electrodes in arrays, and to design the appropriate spatial filters, in particular for the purpose of information extraction regarding dynamics of the human brain’s gamma activity [251].

Deeb [252] analyzed the ECoG and the local field potential signals in patients diagnosed with Parkinson’s disease (PD) to monitor the effects of the disease on their brain activity. It was assumed that there were present low beta and high beta sub-bands ranging between 13–20 Hz and 20–30 Hz. They carried out tests whether this hypothesis applies to all acquired signals in the analyzed data set. The study began with the implementation of the Fourier transform along with the scale-space detection method to test whether the signal exhibits the greatest beta-range behavior. They then extracted the instantaneous frequencies and amplitudes of the signals in the beta range using the empirical wavelet transform (EWT).

Chen et al. [253] proposed a method for extraction and classification of the preactive and the ictal ECoG, based on mutual information together with a supporting vector machine, which has not only high accuracy but also high pace. At the end of the testing, the two methods based on the EMD and ripple were applied as controls.

Hossain et al. [254] investigated phase relationships between the ECoG signals via the Hilbert-Huang transform (HHT) in combination with the EMD. They performed the spatial and temporal filtering of the initial signals, followed by the tuning of the EMD parameters.

Seo et al. [255] proposed a new method based on the dynamic mode decomposition (DMD) to find a significant contrast between the ictal and the interictal patterns in the epileptic EEG data. The DMD-extracted features clearly capture the phase transition of a specific frequency between the channels corresponding with the ictal state and the channel corresponding with the interictal state, such as direct current shift and high-frequency oscillation (HFO). Their study was of a case-study character as it was performed on one patient only, where the ECoG classification tests were carried out at different time intervals, but it showed that the captured phenomenon is a unique pattern, which occurs in the patient’s ictal onset zone.

Ince et al. [256] described in their study an adaptive approach for the classification of the multi-channel ECoG recordings applied for the purpose of the BCI (the invasive one, see [5]). In particular, they implemented a strategy of extracting time plane features from the multi-channel ECoG signals into their proposed approach using divalent undecided wave packet transformation. The transformation of wavelets packets with a double-tree generated a redundant dictionary of functions with different time-frequency resolutions. Rather than evaluating the individual for the discriminant performance of each electrode or candidate element, the proposed approach implemented a wrapper strategy for the selection of a subset of elements from a redundant structured dictionary through evaluation of their combination classification performance. This allowed the algorithm to optimally select non-informative properties originating from different cortical regions and/or time-frequency sites. In Table 3 summary of the most common ECoG signal processing methods was in detail presented.

Finally, the last method worth mentioning in this section is stereotactic electroencephalography (sEEG), which is an invasive method applied for acquiring brain signals. It uses penetrating depth electrodes for electrophysiological brain activity measurement and is most commonly used for epilepsy-generic zones identification. The sEEG-implanted electrodes provide a sparse sampling from deeper brain structures, which cannot be captured by e.g., ECoG. It is also important to mention that the cortical sampling of the sEEG is much sparser than the ECoG, however, it leads to fewer surgical complications than the electrocortical recordings [257,258,259,260]. The sEEG method involves stereotactic orthogonal implantation of depth electrodes (in average 11) [258]. Unfortunately, despite its advantages, it has received very little interest in BCI-related studies [257].

## 5. Discussion

The methods applied for brain signals processing described in this paper are a subjective choice of the authors. This field of science is currently extremely popular, being the subject of interest to a multitude of scientific groups from around the world, therefore it was impossible to describe absolutely all the methods currently used for these signals’ analysis, thus, the authors decided to focus on those they consider as the most effective and/or popular. These methods, introduced and summarized in the previous sections, have still some drawbacks and limitations. Furthermore, many challenges remain in the field of brain-related signals processing. This section lists and discusses these challenges and provides some ideas of the future directions and research possibilities.

### 5.1. Current Challenges

The analysis of brain signals is an extremely difficult task, mainly due to the characteristics of these signals, including their non-stationarity, susceptibility to the occurrence of various types of artifacts or external and internal disturbances, and their low amplitude and frequency range [5,15,16,20]. Moreover, their analysis is challenging due to their non-deterministic nature and absence of specific features such as in the case of ECG signals [5,6]. Although some of the presented methods can suppress considerable amounts and types of noise, they are mostly designed for a given purpose and thus not able to adapt to various environments and conditions. To develop such a robust denoising system, one has to have access to the suitable data set to train and test the algorithms on.

As mentioned in [261], there is the emergence to create a standardized international database, generated using a uniform methodology and data analysis. This would enable us to study individual differences, multi-modal interrelationships and the specificity, or generalizability, of the findings to be examined. For potential practice purposes a very large database with various links with open-source EEG data can be found on GitHub repository in [262].

Due to the Open Science movement, a number of journals have now begun to mandate that authors provide access to the raw data used within the experiments in their papers (e.g., see [263]). Moreover, there are many publicly available EEG databases available, for example, on Physionet.org [264,265]. When choosing a suitable database for their tests, the researchers should keep in mind that the recordings significantly differ based on the purpose the data-set was created for.

To test the filtering methods, there are databases contaminated with motion artifacts [266]. There are also plenty of publicly available databases for assessment of neurological status in sleep [267], or in a specific condition, such as anesthesia [268]. Special attention is also paid to the research of epilepsy, therefore there are also specific databases containing EEG signals during epileptic seizures in both adults [269,270] or pediatric patients [271].

Furthermore, some databases include recordings obtained when the subject was exposed to certain conditions to test his/her cognitive functions or reactions. For example, the subjects could be recorded when performing different motor/imagery tasks [272], before and during the performance of mental arithmetic tasks [273], upon rapid presentation of images through the Rapid Serial Visual Presentation (RSVP) protocol [274], or under the stimulation of flickering lights [275].

### 5.2. Future of Brain Signals’ Analysis

The future of research and innovation development in the area of brain signal processing methods is driven by the following aspects [276]: the increasing expectations and new requirements coming from the clinical practice and other users; the rapid progress in data analysis techniques, such as among the others machine learning, data fusion, or complex network analysis, which enable the development of more and more potential applications for neuroscientific purposes.

The below-mentioned categories appear to be the most promising:

1. *Big Data*: Another future direction is related with big data area, as the big data enable to provide a lot of knowledge and data, necessary for advance methods such as neural networks and deep learning to extract features representing brain functions, mechanisms, or even various disorders or diseases [5,20,277]. Data integration in this field is a very challenging task, as it is necessary for the neuroscientists to measure, share and integrate data [278]. Firstly, it is necessary to have a unified data-set with the same category of subjects, measuring techniques, and protocols applied in it. It is also important to mention the development of other measurement techniques, which provide brain data of better quality, however, techniques such as functional near-infrared spectroscopy (fNIRS) and functional magnetic resonance imaging (fMRI) are expensive and more difficult to operate, and also to analyze. On the other hand, the EEG monitoring provides advantages, such as non-invasiveness, easiness-to-operate, and cost-efficiency [20,279], which makes the EEG particularly suitable for this task [276,280].
2. *Machine learning (ML)*: ML- and pattern-recognition-based methods have been widely applied in neurological signals analysis. They provide new approaches in decoding and enable the characterization of task-related brain states and their extraction from non-informative high-dimensional EEG data. There has been growing interest in the use of ML techniques to analyze EEG [281,282,283]. Multiple studies provided evidence that ML enables efficient extraction of meaningful information even from noisy or contaminated data. The emerging methods of ML, such as transfer learning, reinforcement learning, and ensemble learning, have been gradually used in neuroscience. For example, some new deep neural networks, such as generative adversarial networks and spiking neural networks, have already been applied as powerful tools for EEG decoding, and transfer learning is often adopted by researchers in the area of BCI to increase the accuracy of cross-individual prediction. Also, the BCIs have been widely used to predict behavioral variables and psycho-physiological states from neurological data (particularly EEG) [20,280].
3. *Multi-modality*: Multi-modal neuroimaging can provide a more complimentary picture of the brain and its interaction with other organs. There are many ways to create such a multi-modal system [284]. One of the most commonly applied methods is EEG monitoring, which can be combined with other measurement methods [28,285,286,287,288,289,290,291]:
  - brain imaging techniques, such as MRI and fNIRS;
  - biological signals, such as ECG and EMG;
  - brain stimulation techniques, such as trans-cranial magnetic stimulation (TMS) and trans-cranial direct current stimulation (tDCS).
  Nevertheless, the multi-modal neurological imaging and/or monitoring is associated with specific signal processing and data analyses challenges, such as inter alia [20,292,293,294,295,296,297]:
  - the EEG may obtain artifacts from other biological signals (such as EMG) or be distorted by the noise produced by accompanied devices for imaging (such as MRI) or stimulation (such as TMS). Therefore, signal processing and noise removal techniques play a particularly important role in this field;
  - in terms of data analyses, fusing different neurological modalities to provide complimentary information poses a great issue. Data-driven multivariate methods and machine learning methods can play a role in the analyses of multi-modal brain imaging data.
4. *Real-time implementation*: highly desired in practical applications such as brain-computer interfaces and neurofeedback [298,299]. Real-time applications are very challenging tasks, mostly due to the nature of these signals, however, some promising studies can be seen in: [291,298,300].

## 6. Conclusions

This paper presented the summary and classification of the signals generated by human brain activity, i.e., EEG, EPs, and ECoG. This work is a free continuation of part 1 article dealing with cardiac signals [301]. We summarized the latest articles published within the last 5 years and investigated key information about individual brain signals and associated advanced processing methods with minimization of conference papers and focus on articles published in prestigious journals.

Thanks to the knowledge gained over the years and the combination of the progress in both, the technological and medical solutions, it is possible to monitor the activity of selected parts of the human body using appropriate equipment, which allows obtaining relevant information from biomedical signals [302,303]. The future research lies in discovering additional information in those signals, which has yet been hidden. This will be possible thanks to the rapid development of the acquisition technology, signal processing methods, and analysis.

## Figures and Tables

**Table 1:** Summary of the EEG signal processing methods.

Method	Overall Performance	SNR Improvement	Computational Cost	Real-Time	Implementation Complexity
Filtering	Low	Low	Low	Yes	Simple
WT	Medium	Medium	Medium	Yes	Medium
ICA	Medium	Medium	Medium	Yes	Medium
EMD	Medium	Medium	High	No	Medium
T-F	Medium	Medium	High	No	Complex
Neural Networks	High	Medium	High	Yes	Complex
ANFIS	High	Medium	High	Yes	Complex
Hybrid Methods	High	High	High	Yes	Complex

**Table 2:** Summary of the EP signal processing methods.

Method	Overall Performance	SNR Improvement	Computational Cost	Real-Time	Implementation Complexity
WT	Medium	Medium	Medium	Yes	Medium
PCA	Medium	Medium	Low	Yes	Simple
ICA	Medium	Medium	Medium	Yes	Medium
Hybrid methods	High	High	High	Yes	Complex

**Table 3:** Summary of the ECoG signal processing methods.

Method	Overall Performance	SNR Improvement	Computational Cost	Real-Time	Implementation Complexity
EWT	High	Height	Medium	No	Medium
EMD	Medium	Medium	High	No	Medium
DMD	Medium	Medium	High	Yes	Medium
T-F	High	Medium	High	No	Complex

## References

[1] Von Neumann J. (2012). The Computer and the Brain.

[2] Gao S., Wang Y., Gao X., Hong B. (2014). Visual and auditory brain–computer interfaces. IEEE Trans. Biomed. Eng..

[3] Chandra P. (2017). A Survey on Deep Learning its Architecture and Various Applications. Asia Pac. J. Neural Netw. Appl..

[4] Swanson L.W. (2012). Brain Architecture: Understanding the Basic Plan.

[5] Kawala-Janik A. (2013). Efficiency Evaluation of External Environments Control Using Bio-Signals. Ph.D. Thesis.

[6] Gao X., Xu D., Cheng M., Gao S. (2003). A BCI-based environmental controller for the motion-disabled. IEEE Trans. Neural Syst. Rehabil. Eng..

[7] Kiloh L.G., McComas A.J., Osselton J.W. (1972). Clinical Electroencephalography.

[8] Millett D. (2001). Hans Berger: From Psychic Energy to the EEG. Perspect. Biol. Med..

[9] Coenen A., Zayachkivska O. (2013). Adolf Beck: A Pioneer in Electroencephalography in between Richard Caton and Hans Berger. Adv. Cogn. Psychol..

[10] Jurcak V., Tsuzuki D., Dan I. (2007). 10/20, 10/10, and 10/5 Systems Revisited: Their Validity as Relative Head-Surface-Based Positioning Systems. NeuroImage.

[11] Ball T., Kern M., Mutschler I., Aertsen A., Schulze-Bonhage A. (2009). Signal Quality of Simultaneously Recorded Invasive and Non-Invasive EEG. NeuroImage.

[12] Acharya J.N., Acharya V.J. (2019). Overview of EEG montages and principles of localization. J. Clin. Neurophysiol..

[13] Sazgar M., Young M.G. (2019). Overview of EEG, electrode placement, and montages. Absolute Epilepsy and EEG Rotation Review.

[14] Kutluay E., Kalamangalam G.P. (2019). Montages for Noninvasive EEG Recording. J. Clin. Neurophysiol. Off. Publ. Am. Electroencephalogr. Soc..

[15] Kawala-Janik A., Pelc M., Podpora M. (2015). Method for EEG Signals Pattern Recognition in Embedded Systems. Elektron. Elektrotechnika.

[16] Kawala-Sterniuk A., Podpora M., Pelc M., Blaszczyszyn M., Gorzelanczyk E.J., Martinek R., Ozana S. (2020). Comparison of Smoothing Filters in Analysis of EEG Data for the Medical Diagnostics Purposes. Sensors.

[17] Tomasz R. (2015). Brain–Robot and Speller Interfaces Using Spatial Multisensory Brain-Computer Interface Paradigms. Front. Comput. Neurosci..

[18] Allison B.Z., Neuper C., Tan D.S., Nijholt A. (2010). Could Anyone Use a BCI?. Brain-Computer Interfaces.

[19] Cichocki A., Washizawa Y., Rutkowski T., Bakardjian H., Phan A.H., Choi S., Lee H., Zhao Q., Zhang L., Li Y. (2008). Noninvasive BCIs: Multiway Signal-Processing Array Decompositions. Computer.

[20] Kawala-Sterniuk A., Browarska N., Al-Bakri A., Pelc M., Zygarlicki J., Sidikova M., Martinek R., Gorzelanczyk E.J. (2021). Summary of over Fifty Years with Brain-Computer Interfaces—A Review. Brain Sci..

[21] Miller K.J., Hermes D., Staff N.P. (2020). The current state of electrocorticography-based brain–computer interfaces. Neurosurg. Focus.

[22] Oostenveld R., Praamstra P. (2001). The Five Percent Electrode System for High-Resolution EEG and ERP Measurements. Clin. Neurophysiol..

[23] Ferree T.C., Luu P., Russell G.S., Tucker D.M. (2001). Scalp Electrode Impedance, Infection Risk, and EEG Data Quality. Clin. Neurophysiol..

[24] Gopan K.G., Prabhu S.S., Sinha N. (2020). Sleep EEG Analysis Utilizing Inter-Channel Covariance Matrices. Biocybern. Biomed. Eng..

[25] Banville H., Chehab O., Hyvärinen A., Engemann D.A., Gramfort A. (2020). Uncovering the Structure of Clinical EEG Signals with Self-Supervised Learning. arXiv.

[26] Rangayyan R.M. (2015). Biomedical Signal Analysis.

[27] Penhaker M., Augustynek M. (2013). Zdravotnické Elektrické Přístroje 1.

[28] Nyni K., Vincent L.K., Varghese L., Liya V., Johny A.N., Yesudas C. (2017). Wireless health monitoring system for ECG, EMG and EEG detecting. Proceedings of the 2017 International Conference on Innovations in Information, Embedded and Communication Systems (ICIIECS).

[29] Fink A., Grabner R., Neuper C., Neubauer A. (2005). EEG Alpha Band Dissociation with Increasing Task Demands. Cogn. Brain Res..

[30] Chervin R.D., Burns J.W., Subotic N.S., Roussi C., Thelen B., Ruzicka D.L. (2004). Correlates of Respiratory Cycle-Related EEG Changes in Children with Sleep-Disordered Breathing. Sleep.

[31] van Albada S.J., Robinson P.A. (2013). Relationships between Electroencephalographic Spectral Peaks Across Frequency Bands. Front. Hum. Neurosci..

[32] Bruce E.N. (2001). Biomedical Signal Processing and Signal Modeling.

[33] Sharma M., Patel S., Acharya U.R. (2020). Automated Detection of Abnormal EEG Signals Using Localized Wavelet Filter Banks. Pattern Recognit. Lett..

[34] Fernandez-Baca Vaca G., Park J.T. (2020). Focal EEG Abnormalities and Focal Ictal Semiology in Generalized Epilepsy. Seizure.

[35] Harris L., Angus-Leppan H. (2020). Epilepsy: Diagnosis, Classification and Management. Medicine.

[36] Dalle Ave A.L., Bernat J.L. (2020). Inconsistencies Between the Criterion and Tests for Brain Death. J. Intensive Care Med..

[37] Emmady P.D., Anilkumar A.C. (2020). EEG, Abnormal Waveforms. StatPearls.

[38] Gurrala V., Yarlagadda P., Koppireddi P. (2021). Detection of Sleep Apnea Based on the Analysis of Sleep Stages Data Using Single Channel EEG. Trait. du Signal.

[39] Jain S.V., Dye T., Kedia P. (2019). Value of combined video EEG and polysomnography in clinical management of children with epilepsy and daytime or nocturnal spells. Seizure.

[40] Melia U., Guaita M., Vallverdú M., Embid C., Vilaseca I., Salamero M., Santamaria J. (2015). Mutual information measures applied to EEG signals for sleepiness characterization. Med. Eng. Phys..

[41] Buettner R., Fuhrmann J., Kolb L. (2019). Towards high-performance differentiation between Narcolepsy and Idiopathic Hypersomnia in 10 minute EEG recordings using a Novel Machine Learning Approach. Proceedings of the 2019 IEEE International Conference on E-health Networking, Application & Services (HealthCom).

[42] Sarilar A.C., Ismailogullari S., Yilmaz R., Erdogan F.F., Per H. (2019). Electroencephalogram abnormalities in patients with NREM parasomnias. Sleep Med..

[43] Mishra S., Birok R. (2021). Literature review: Sleep stage classification based on EEG signals using artificial intelligence technique. Recent Trends in Communication and Electronics.

[44] Sunwoo J.S., Cha K.S., Byun J.I., Jun J.S., Kim T.J., Shin J.W., Lee S.T., Jung K.H., Park K.I., Chu K. (2020). NREM sleep EEG oscillations in idiopathic REM sleep behavior disorder: A study of sleep spindles and slow oscillations. Sleep.

[45] Nuwer M.R., Jordan S.E., Ahn S.S. (1987). Evaluation of Stroke Using EEG Frequency Analysis and Topographic Mapping. Neurology.

[46] Juhasz Z. (2020). Quantitative Cost Comparison of On-Premise and Cloud Infrastructure Based EEG Data Processing. Clust. Comput..

[47] Kapgate D. (2020). Future of EEG Based Hybrid Visual Brain Computer Interface Systems in Rehabilitation of People with Neurological Disorders. Int. Res. J. Adv. Sci. Hub (IRJASH).

[48] Asadzadeh S., Yousefi Rezaii T., Beheshti S., Delpak A., Meshgini S. (2020). A Systematic Review of EEG Source Localization Techniques and Their Applications on Diagnosis of Brain Abnormalities. J. Neurosci. Methods.

[49] Maidana Capitán M., Cámpora N., Sigvard C.S., Kochen S., Samengo I. (2020). Time- and Frequency-Resolved Covariance Analysis for Detection and Characterization of Seizures from Intracraneal EEG Recordings. Biol. Cybern..

[50] Giuliano L., Mainieri G., Cicero C.E., Battaglia G., Guccione A., Salomone S., Drago F., Nicoletti A., Sofia V., Zappia M. (2020). Parasomnias, Sleep-Related Movement Disorders and Physiological Sleep Variants in Focal Epilepsy: A Polysomnographic Study. Seizure.

[51] Savadkoohi M., Oladunni T., Thompson L. (2020). A Machine Learning Approach to Epileptic Seizure Prediction Using Electroencephalogram (EEG) Signal. Biocybern. Biomed. Eng..

[52] Reus E.E., Visser G.H., Cox F.M. (2020). Using Sampled Visual EEG Review in Combination with Automated Detection Software at the EMU. Seizure.

[53] Al-Bakri A.F., Villamar M.F., Haddix C., Bensalem-Owen M., Sunderam S. (2018). Noninvasive seizure prediction using autonomic measurements in patients with refractory epilepsy. Proceedings of the 2018 40th Annual International Conference of the IEEE Engineering in Medicine and Biology Society (EMBC).

[54] Cox F., Reus E., Widman G., Zwemmer J., Visser G. (2020). Epilepsy Monitoring Units Can Be Safe Places; a Prospective Study in a Large Cohort. Epilepsy Behav..

[55] Duy P.Q., Krauss G.L., Crone N.E., Ma M., Johnson E.L. (2020). Antiepileptic Drug Withdrawal and Seizure Severity in the Epilepsy Monitoring Unit. Epilepsy Behav..

[56] Askamp J., van Putten M.J. (2014). Mobile EEG in epilepsy. Int. J. Psychophysiol..

[57] Gilliam F., Kuzniecky R., Faught E. (1999). Ambulatory EEG monitoring. J. Clin. Neurophysiol..

[58] Elger C.E., Hoppe C. (2018). Diagnostic challenges in epilepsy: Seizure under-reporting and seizure detection. Lancet Neurol..

[59] Brunnhuber F., Slater J., Goyal S., Amin D., Thorvardsson G., Freestone D.R., Richardson M.P. (2020). Past, Present and Future of Home video-electroencephalographic telemetry: A review of the development of in-home video-electroencephalographic recordings. Epilepsia.

[60] Mohammed N.S., Al-Mamoori M.J., Albermani A., AbudAlameer W.R., Abbas F.N. (2020). Electroencephalogram and Visual Evoked Potential Changes in Patients with Primary Headaches. Indian J. Forensic Med. Toxicol..

[61] Somaiya S. Electroencephalogram (EEG): Meaning, Sources and Significance. 2016. https://www.biologydiscussion.com/human-physiology/electroencephalogram/electroencephalogram-eeg-meaning-sources-and-significance/62944?fbclid=IwAR0RNKnj2dBNPUABXEtPIxdoWuZIAFLOQYgW8vbqD7PYyrvzah22WGc9xhY.

[62] Tatum W.O. (2014). Handbook of EEG Interpretation.

[63] Chernecky C.C., Berger B.J. (2013). Laboratory Tests and Diagnostic Procedures.

[64] Cuellar M., Harkrider A., Jenson D., Thornton D., Bowers A., Saltuklaroglu T. (2016). Time–Frequency Analysis of the EEG Mu Rhythm as a Measure of Sensorimotor Integration in the Later Stages of Swallowing. Clin. Neurophysiol..

[65] Martins N.R., Angelica A., Chakravarthy K., Svidinenko Y., Boehm F.J., Opris I., Lebedev M.A., Swan M., Garan S.A., Rosenfeld J.V. (2019). Human brain/cloud interface. Front. Neurosci..

[66] Cecotti H. (2011). Spelling with non-invasive brain–computer Interfaces—Current and future trends. J. Physiol.-Paris.

[67] Yu X., Qi W. A user study of wearable EEG headset products for emotion analysis. Proceedings of the 2018 International Conference on Algorithms, Computing and Artificial Intelligence.

[68] Das R., Chatterjee D., Das D., Sinharay A., Sinha A. (2014). Cognitive load measurement—A methodology to compare low cost commercial eeg devices. Proceedings of the 2014 International conference on advances in computing, communications and informatics (ICACCI).

[69] Stytsenko K., Jablonskis E., Prahm C. Evaluation of consumer EEG device Emotiv EPOC. Proceedings of the MEi: CogSci Conference 2011.

[70] Frey J. Comparison of a consumer grade EEG amplifier with medical grade equipment in BCI applications. Proceedings of the International BCI Meeting.

[71] Katona J., Kovari A. (2018). The evaluation of bci and pebl-based attention tests. Acta Polytech. Hung..

[72] Frey J. (2016). Comparison of an open-hardware electroencephalography amplifier with medical grade device in brain-computer interface applications. arXiv.

[73] Ekandem J.I., Davis T.A., Alvarez I., James M.T., Gilbert J.E. (2012). Evaluating the ergonomics of BCI devices for research and experimentation. Ergonomics.

[74] Sugiono S., Putra A.S., Fanani A.A., Cahyawati A.N., Oktavianty O. (2021). A New Concept of Product Design by Involving Emotional Factors Using Eeg: A Case Study of Xomputer Mouse Design. Acta Neuropsychol..

[75] Lacko D., Vleugels J., Fransen E., Huysmans T., De Bruyne G., Van Hulle M.M., Sijbers J., Verwulgen S. (2017). Ergonomic design of an EEG headset using 3D anthropometry. Appl. Ergon..

[76] Rogers J.M., Jensen J., Valderrama J.T., Johnstone S.J., Wilson P.H. (2021). Single-channel EEG measurement of engagement in virtual rehabilitation: A validation study. Virtual Real..

[77] Zhang J., Yao R., Ge W., Gao J. (2020). Orthogonal convolutional neural networks for automatic sleep stage classification based on single-channel EEG. Comput. Methods Programs Biomed..

[78] Ko L.W., Komarov O., Lai W.K., Liang W.G., Jung T.P. (2020). Eyeblink recognition improves fatigue prediction from single-channel forehead EEG in a realistic sustained attention task. J. Neural Eng..

[79] Eldele E., Chen Z., Liu C., Wu M., Kwoh C.K., Li X., Guan C. (2021). An Attention-Based Deep Learning Approach for Sleep Stage Classification With Single-Channel EEG. IEEE Trans. Neural Syst. Rehabil. Eng..

[80] Kaur J., Kaur A. (2015). A review on analysis of EEG signals. Proceedings of the 2015 International Conference on Advances in Computer Engineering and Applications.

[81] Lee S., Buchsbaum M.S. (1987). Topographic Mapping of EEG Artifacts. Clin. EEG (Electroencephalogr.).

[82] Durka P., Klekowicz H., Blinowska K., Szelenberger W., Niemcewicz S. (2003). A Simple System for Detection of EEG Artifacts in Polysomnographic Recordings. IEEE Trans. Biomed. Eng..

[83] Moretti D. (2003). Computerized Processing of EEG–EOG–EMG Artifacts for Multi-Centric Studies in EEG Oscillations and Event-Related Potentials. Int. J. Psychophysiol..

[84] Correa A.G., Laciar E., Patiño H., Valentinuzzi M. (2007). Artifact removal from EEG signals using adaptive filters in cascade. J. Phys. Conf. Ser..

[85] Leske S., Dalal S.S. (2019). Reducing power line noise in EEG and MEG data via spectrum interpolation. Neuroimage.

[86] La Rosa A.B., Pereira P.T., Ücker P., Paim G., da Costa E.A., Bampi S., Almeida S. (2021). Exploring NLMS-Based Adaptive Filter Hardware Architectures for Eliminating Power Line Interference in EEG Signals. Circuits Syst. Signal Process..

[87] Reddy A.G., Narava S. (2013). Artifact removal from EEG signals. Int. J. Comput. Appl..

[88] Qian X., Xu Y.P., Li X. (2005). A CMOS continuous-time low-pass notch filter for EEG systems. Analog Integr. Circuits Signal Process..

[89] Saini M., Satija U., Upadhayay M.D. (2020). Effective Automated Method for Detection and Suppression of Muscle Artefacts from Single-Channel EEG Signal. Healthc. Technol. Lett..

[90] Shah S.A.A., Zhang L., Bais A. (2020). Dynamical System Based Compact Deep Hybrid Network for Classification of Parkinson Disease Related EEG Signals. Neural Netw..

[91] Silva G., Alves M., Cunha R., Bispo B.C., Rodrigues P.M. (2020). Parkinson Disease Early Detection Using EEG Channels Cross-Correlation. Int. J. Appl. Eng. Res..

[92] Noureddin B., Lawrence P.D., Birch G.E. (2012). Online Removal of Eye Movement and Blink EEG Artifacts Using a High-Speed Eye Tracker. IEEE Trans. Biomed. Eng..

[93] Jansen M., White T.P., Mullinger K.J., Liddle E.B., Gowland P.A., Francis S.T., Bowtell R., Liddle P.F. (2012). Motion-Related Artefacts in EEG Predict Neuronally Plausible Patterns of Activation in fMRI Data. NeuroImage.

[94] Abbaspour H., Mehrshad N., Razavi S.M., Mesin L. (2019). Artefacts Removal to Detect Visual Evoked Potentials in Brain Computer Interface Systems. J. Biomimetics Biomater. Biomed. Eng..

[95] Diykh M., Li Y., Abdulla S. (2020). EEG Sleep Stages Identification Based on Weighted Undirected Complex Networks. Comput. Methods Programs Biomed..

[96] Jiao Y., Deng Y., Luo Y., Lu B.L. (2020). Driver Sleepiness Detection from EEG and EOG Signals Using GAN and LSTM Networks. Neurocomputing.

[97] Wang Y., Xu G., Zhang S., Luo A., Li M., Han C. (2017). EEG Signal Co-Channel Interference Suppression Based on Image Dimensionality Reduction and Permutation Entropy. Signal Process..

[98] Benbadis S.R. EEG Artifacts: Overview, Physiologic Artifacts, Extraphysiologic Artifacts. 2019. https://emedicine.medscape.com/article/1140247-overview.

[99] Tandle A., Jog N. (2015). Classification of Artefacts in EEG Signal Recordings and Overview of Removing Techniques. Int. J. Comput. Appl..

[100] Cichocki A., Rutkowski T., Siwek K. Blind Signal Extraction of Signals with Specified Frequency Band. Proceedings of the 12th IEEE Workshop on Neural Networks for Signal Processing.

[101] Gallego-Jutglà E., Solé-Casals J., Rutkowski T.M., Cichocki A. Application of Multivariate Empirical Mode Decomposition for Cleaning Eye Blinks Artifacts from EEG Signals. Proceedings of the International Conference on Neural Computation Theory and Applications (Special Session on Challenges in Neuroengineering-2011).

[102] Rakhmatulin I. (2020). Review of EEG Feature Selection by Neural Networks. Int. J. Sci. Bus..

[103] Baig M.Z., Aslam N., Shum H.P.H. (2020). Filtering Techniques for Channel Selection in Motor Imagery EEG Applications: A Survey. Artif. Intell. Rev..

[104] Higashi H., Rutkowski T.M., Tanaka T., Tanaka Y. Smoothing of xDAWN Spatial Filters for Robust Extraction of Event-Related Potentials. Proceedings of the 2016 Asia-Pacific Signal and Information Processing Association Annual Summit and Conference (APSIPA).

[105] Schlögl A., Anderer P., Roberts S.J., Pregenzer M., Pfurtscheller G. Artefact Detection in Sleep EEG by the Use of Kalman Filtering. Proceedings of the EMBEC’99.

[106] Li J., Chan S.C., Liu Z., Chang C. (2020). A Novel Adaptive Fading Kalman Filter-Based Approach to Time-Varying Brain Spectral/Connectivity Analyses of Event-Related EEG Signals. IEEE Access.

[107] Qi F., Wu W., Yu Z.L., Gu Z., Wen Z., Yu T., Li Y. (2020). Spatiotemporal-Filtering-Based Channel Selection for Single-Trial EEG Classification. IEEE Trans. Cybern..

[108] Ahmed O.I., Yassin H.M., Said L.A., Psychalinos C., Radwan A.G. (2020). Implementation and Analysis of Tunable Fractional-Order Band-Pass Filter of Order 2*α*. AEU—Int. J. Electron. Commun..

[109] Baranowski J., Piatek P. (2017). Fractional Band-Pass Filters: Design, Implementation and Application to EEG Signal Processing. J. Circuits Syst. Comput..

[110] Baranowski J., Bauer W., Zagorowska M., Piatek P. (2016). On Digital Realizations of Non-Integer Order Filters. Circuits Syst. Signal Process..

[111] Baranowski J., Pauluk M., Tutaj A. (2017). Analog Realization of Fractional Filters: Laguerre Approximation Approach. AEU—Int. J. Electron. Commun..

[112] Elwy O., Abdelaty A., Said L., Radwan A. (2020). Fractional Calculus Definitions, Approximations, and Engineering Applications. J. Eng. Appl. Sci..

[113] Nagabushanam P., George S.T., Dolly D.R.J., Radha S. (2020). Artifact Cleaning of Motor Imagery EEG by Statistical Features Extraction Using Wavelet Families. Int. J. Circuit Theory Appl..

[114] Bhati D., Sharma M., Pachori R.B., Gadre V.M. (2017). Time—Frequency Localized Three-Band Biorthogonal Wavelet Filter Bank Using Semidefinite Relaxation and Nonlinear Least Squares with Epileptic Seizure EEG Signal Classification. Digit. Signal Process..

[115] Bhattacharyya A., Sharma M., Pachori R.B., Sircar P., Acharya U.R. (2018). A Novel Approach for Automated Detection of Focal EEG Signals Using Empirical Wavelet Transform. Neural Comput. Appl..

[116] Mamun M., Al-Kadi M., Marufuzzaman M. (2013). Effectiveness of Wavelet Denoising on Electroencephalogram Signals. J. Appl. Res. Technol..

[117] Yavuz E., Aydemir O. Olfaction Recognition by EEG Analysis Using Wavelet Transform Features. Proceedings of the 2016 International Symposium on INnovations in Intelligent SysTems and Applications (INISTA).

[118] Ablin P., Cardoso J.F., Gramfort A. (2020). Spectral Independent Component Analysis with Noise Modeling for M/EEG Source Separation. arXiv.

[119] Devulapalli S.P., Chanamallu S.R., Kodati S.P. (2020). A Hybrid ICA Kalman Predictor Algorithm for Ocular Artifacts Removal. Int. J. Speech Technol..

[120] Hsu S.H., Mullen T.R., Jung T.P., Cauwenberghs G. (2015). Real-time adaptive EEG source separation using online recursive independent component analysis. IEEE Trans. Neural Syst. Rehabil. Eng..

[121] Chang C.Y., Hsu S.H., Pion-Tonachini L., Jung T.P. (2019). Evaluation of artifact subspace reconstruction for automatic artifact components removal in multi-channel EEG recordings. IEEE Trans. Biomed. Eng..

[122] Shan X., Yang E.H., Zhou J., Chang V.W.C. (2018). Human-Building Interaction under Various Indoor Temperatures through Neural-Signal Electroencephalogram (EEG) Methods. Build. Environ..

[123] Corradino C., Bucolo M. Automatic Preprocessing of EEG Signals in Long Time Scale. Proceedings of the 2015 37th Annual International Conference of the IEEE Engineering in Medicine and Biology Society (EMBC).

[124] Li W., Shen Y., Zhang J., Huang X., Chen Y., Ge Y. (2018). Common Interferences Removal from Dense Multichannel EEG Using Independent Component Decomposition. Comput. Math. Methods Med..

[125] Arnin J., Kahani D., Lakany H., Conway B.A. Evaluation of Different Signal Processing Methods in Time and Frequency Domain for Brain-Computer Interface Applications. Proceedings of the 2018 40th Annual International Conference of the IEEE Engineering in Medicine and Biology Society (EMBC).

[126] Chaudhary U., Mrachacz-Kersting N., Birbaumer N. (2020). Neuropsychological and neurophysiological aspects of brain-computer-interface (BCI) control in paralysis. J. Physiol..

[127] Borgheai S.B., McLinden J., Zisk A.H., Hosni S.I., Deligani R.J., Abtahi M., Mankodiya K., Shahriari Y. (2020). Enhancing Communication for People in Late-Stage ALS Using an fNIRS-Based BCI System. IEEE Trans. Neural Syst. Rehabil. Eng..

[128] Piccione F., Giorgi F., Tonin P., Priftis K., Giove S., Silvoni S., Palmas G., Beverina F. (2006). P300-Based Brain Computer Interface: Reliability and Performance in Healthy and Paralysed Participants. Clin. Neurophysiol..

[129] Khan O.I., Farooq F., Akram F., Choi M.T., Han S.M., Kim T.S. (2012). Robust Extraction of P300 Using Constrained ICA for BCI Applications. Med. Biol. Eng. Comput..

[130] Rashid M., Sulaiman N., Mustafa M., Khatun S., Bari B.S., Hasan M.J., Kasruddin Nasir A.N., Ahmad M.A., Najib M.S., Abdul Wahab Y., Othman N.A., Abd Ghani N.M., Irawan A., Khatun S., Raja Ismail R.M.T., Saari M.M. (2020). Recent Trends and Open Challenges in EEG Based Brain-Computer Interface Systems. InECCE2019.

[131] Pion-Tonachini L., Hsu S.H., Makeig S., Jung T.P., Cauwenberghs G. (2015). Real-time eeg source-mapping toolbox (rest): Online ica and source localization. Proceedings of the 2015 37th Annual International Conference of the IEEE Engineering in Medicine and Biology Society (EMBC).

[132] Flandrin P., Rilling G., Goncalves P. (2004). Empirical Mode Decomposition as a Filter Bank. IEEE Signal Process. Lett..

[133] Amo C., De Santiago L., Barea R., López-Dorado A., Boquete L. (2017). Analysis of Gamma-Band Activity from Human EEG Using Empirical Mode Decomposition. Sensors.

[134] Chen S.J., Peng C.J., Chen Y.C., Hwang Y.R., Lai Y.S., Fan S.Z., Jen K.K. (2016). Comparison of FFT and Marginal Spectra of EEG Using Empirical Mode Decomposition to Monitor Anesthesia. Comput. Methods Programs Biomed..

[135] Gaur P., Pachori R.B., Wang H., Prasad G. (2018). A Multi-Class EEG-Based BCI Classification Using Multivariate Empirical Mode Decomposition Based Filtering and Riemannian Geometry. Expert Syst. Appl..

[136] Rutkowski T.M., Mandic D.P., Cichocki A., Przybyszewski A.W. (2010). EMD Approach to Multichannel EEG Data—The Amplitude and Phase Components Clustering Analysis. J. Circuits Syst. Comput..

[137] Molla M.K.I., Tanaka T., Rutkowski T.M., Cichocki A. Separation of EOG Artifacts from EEG Signals Using Bivariate EMD. Proceedings of the 2010 IEEE International Conference on Acoustics, Speech and Signal Processing.

[138] Maimon N.B., Molcho L., Intrator N., Lamy D. (2020). Single-Channel EEG Features during n-Back Task Correlate with Working Memory Load. arXiv.

[139] Leite N.M.N., Pereira E.T., Gurjão E.C., Veloso L.R. (2018). Deep convolutional autoencoder for eeg noise filtering. Proceedings of the 2018 IEEE International Conference on Bioinformatics and Biomedicine (BIBM).

[140] Yang B., Duan K., Fan C., Hu C., Wang J. (2018). Automatic ocular artifacts removal in EEG using deep learning. Biomed. Signal Process. Control.

[141] Jafarifarmand A., Badamchizadeh M.A. (2013). Artifacts removal in EEG signal using a new neural network enhanced adaptive filter. Neurocomputing.

[142] Cowan H., Daryanavard S., Porr B., Dahiya R. (2020). A real-time noise cancelling EEG electrode employing Deep Learning. arXiv.

[143] Quej V.H., Almorox J., Arnaldo J.A., Saito L. (2017). ANFIS, SVM and ANN soft-computing techniques to estimate daily global solar radiation in a warm sub-humid environment. J. Atmos. Sol.-Terr. Phys..

[144] Jang J.S. (1993). ANFIS: Adaptive-Network-Based Fuzzy Inference System. IEEE Trans. Syst. Man Cybern..

[145] Chen W., Wang Z., Lao K.F., Wan F. (2014). Ocular artifact removal from EEG using ANFIS. Proceedings of the 2014 IEEE International Conference on Fuzzy Systems (FUZZ-IEEE).

[146] Pereira L.F., Patil S.A., Mahadeshwar C.D., Mishra I., D’Souza L. (2016). Artifact removal from EEG using ANFIS-GA. Proceedings of the 2016 Online International Conference on Green Engineering and Technologies (IC-GET).

[147] Akhtar M.T., James C.J. (2009). Focal artifact removal from ongoing EEG–a hybrid approach based on spatially-constrained ICA and wavelet de-noising. Proceedings of the 2009 Annual International Conference of the IEEE Engineering in Medicine and Biology Society.

[148] Akhtar M.T., Mitsuhashi W., James C.J. (2012). Employing spatially constrained ICA and wavelet denoising, for automatic removal of artifacts from multichannel EEG data. Signal Process..

[149] Peng H., Hu B., Shi Q., Ratcliffe M., Zhao Q., Qi Y., Gao G. (2013). Removal of Ocular Artifacts in EEG—An Improved Approach Combining DWT and ANC for Portable Applications. IEEE J. Biomed. Health Inform..

[150] Maddirala A., Shaik R.A. (2016). Removal of EOG Artifacts from Single Channel EEG Signals Using Combined Singular Spectrum Analysis and Adaptive Noise Canceler. IEEE Sens. J..

[151] Nguyen H.A.T., Le T.H., Bui T.D. (2020). A Deep Wavelet Sparse Autoencoder Method for Online and Automatic Electrooculographical Artifact Removal. Neural Comput. Appl..

[152] Kose M.R., Ahirwal M.K., Janghel R.R. (2020). Descendant Adaptive Filter to Remove Different Noises from ECG Signals. Int. J. Biomed. Eng. Technol..

[153] Kaya I. (2019). A Brief Summary of EEG Artifact Handling. arXiv.

[154] Jafarifarmand A., Badamchizadeh M.A., Khanmohammadi S., Nazari M.A., Tazehkand B.M. (2017). Real-time ocular artifacts removal of EEG data using a hybrid ICA-ANC approach. Biomed. Signal Process. Control.

[155] Torse D.A., Desai V.V. Design of Adaptive EEG Preprocessing Algorithm for Neurofeedback System. Proceedings of the 2016 International Conference on Communication and Signal Processing (ICCSP).

[156] Rejer I., Górski P., Rutkowski L., Scherer R., Korytkowski M., Pedrycz W., Tadeusiewicz R., Zurada J.M. (2019). A Multi-Filtering Algorithm for Applying ICA in a Low-Channel EEG. Artificial Intelligence and Soft Computing.

[157] Dimitriadis S.I., Marimpis A.D. (2018). Enhancing performance and bit rates in a brain–computer interface system with phase-to-amplitude cross-frequency coupling: Evidences from traditional c-VEP, Fast c-VEP, and SSVEP designs. Front. Neuroinform..

[158] Cohen M.X. (2008). Assessing transient cross-frequency coupling in EEG data. J. Neurosci. Methods.

[159] Knyazev G.G., Savostyanov A.N., Bocharov A.V., Tamozhnikov S.S., Kozlova E.A., Leto I.V., Slobodskaya H.R. (2019). Cross-frequency coupling in developmental perspective. Front. Hum. Neurosci..

[160] Dimitriadis S.I., Laskaris N.A., Bitzidou M.P., Tarnanas I., Tsolaki M.N. (2015). A novel biomarker of amnestic MCI based on dynamic cross-frequency coupling patterns during cognitive brain responses. Front. Neurosci..

[161] Dimitriadis S.I., Laskaris N.A., Simos P.G., Fletcher J.M., Papanicolaou A.C. (2016). Greater repertoire and temporal variability of cross-frequency coupling (CFC) modes in resting-state neuromagnetic recordings among children with reading difficulties. Front. Hum. Neurosci..

[162] Cohen W.R., Hayes-Gill B. (2014). Influence of Maternal Body Mass Index on Accuracy and Reliability of External Fetal Monitoring Techniques. Acta Obstet. et Gynecol. Scand..

[163] Jirsa V., Müller V. (2013). Cross-frequency coupling in real and virtual brain networks. Front. Comput. Neurosci..

[164] Sakkalis V. (2011). Review of advanced techniques for the estimation of brain connectivity measured with EEG/MEG. Comput. Biol. Med..

[165] Haufe S., Nikulin V.V., Müller K.R., Nolte G. (2013). A critical assessment of connectivity measures for EEG data: A simulation study. Neuroimage.

[166] Ullah I., Hussain M., Aboalsamh H. (2018). An automated system for epilepsy detection using EEG brain signals based on deep learning approach. Expert Syst. Appl..

[167] Nigam V.P., Graupe D. (2004). A neural-network-based detection of epilepsy. Neurol. Res..

[168] Oh S.L., Hagiwara Y., Raghavendra U., Yuvaraj R., Arunkumar N., Murugappan M., Acharya U.R. (2020). A deep learning approach for Parkinson’s disease diagnosis from EEG signals. Neural Comput. Appl..

[169] Merlin Praveena D., Angelin Sarah D., Thomas George S. (2020). Deep learning techniques for EEG signal applications—A review. IETE J. Res..

[170] Guo L., Rivero D., Dorado J., Rabunal J.R., Pazos A. (2010). Automatic epileptic seizure detection in EEGs based on line length feature and artificial neural networks. J. Neurosci. Methods.

[171] Zhang X., Yao L., Wang X., Monaghan J.J., Mcalpine D., Zhang Y. (2020). A survey on deep learning-based non-invasive brain signals: Recent advances and new frontiers. J. Neural Eng..

[172] Zhang X., Yao L., Zhang S., Kanhere S., Sheng M., Liu Y. (2018). Internet of Things meets brain–computer interface: A unified deep learning framework for enabling human-thing cognitive interactivity. IEEE Internet Things J..

[173] Islam M.R., Moni M.A., Islam M.M., Rashed-Al-Mahfuz M., Islam M.S., Hasan M.K., Hossain M.S., Ahmad M., Uddin S., Azad A. (2021). Emotion Recognition From EEG Signal Focusing on Deep Learning and Shallow Learning Techniques. IEEE Access.

[174] Luo J., Gao X., Zhu X., Wang B., Lu N., Wang J. (2020). Motor Imagery EEG Classification Based on Ensemble Support Vector Learning. Comput. Methods Programs Biomed..

[175] Sabeti M., Boostani R., Moradi E. (2020). Event Related Potential (ERP) as a Reliable Biometric Indicator: A Comparative Approach. Array.

[176] Markand O.N. (2020). Basic Techniques of Evoked Potential Recording. Clinical Evoked Potentials.

[177] Kumar A., Anand S., Yaddanapudi L.N. (2006). Comparison of Auditory Evoked Potential Parameters for Predicting Clinically Anaesthetized State. Acta Anaesthesiol. Scand..

[178] Cruccu G., Aminoff M., Curio G., Guerit J., Kakigi R., Mauguiere F., Rossini P., Treede R.D., Garcia-Larrea L. (2008). Recommendations for the Clinical Use of Somatosensory-Evoked Potentials. Clin. Neurophysiol..

[179] Lueders H., Lesser R.P., Hahn J., Dinner D.S., Klem G. (1983). Cortical Somatosensory Evoked Potentials in Response to Hand Stimulation. J. Neurosurg..

[180] Cracco R.Q., Cracco J.B. (1976). Somatosensory Evoked Potential in Man: Far Field Potentials. Electroencephalogr. Clin. Neurophysiol..

[181] Chiappa K.H. (1997). Evoked Potentials in Clinical Medicine.

[182] Najarian K. (2016). Biomedical Signal and Image Processing.

[183] Cook M.L., Varela R.A., Goldstein J.D., McCulloch S.D., Bossart G.D., Finneran J.J., Houser D., Mann D.A. (2006). Beaked whale auditory evoked potential hearing measurements. J. Comp. Physiol. A.

[184] Borges L.R., Donadon C., Sanfins M.D., Valente J.P., Paschoal J.R., Colella-Santos M.F. (2020). The effects of otitis media with effusion on the measurement of auditory evoked potentials. Int. J. Pediatr. Otorhinolaryngol..

[185] Saha S., Mamun K.A., Ahmed K.I.U., Mostafa R., Naik G.R., Darvishi S., Khandoker A.H., Baumert M. (2021). Progress in Brain Computer Interface: Challenges and Potentials. Front. Syst. Neurosci..

[186] Welschehold S., Boor S., Reuland K., Thömke F., Kerz T., Reuland A., Beyer C., Gartenschläger M., Wagner W., Giese A. (2012). Technical Aids in the Diagnosis of Brain Death. Dtsch. Aerzteblatt Online.

[187] Picton T.W. (2010). Human Auditory Evoked Potentials.

[188] Capitanio L., Jensen E.W., Filligoi G.C., Makovec B., Gagliardi M., Henneberg S.W., Lindholm P., Cerutti S. (1997). On-Line Analysis of AEP and EEG for Monitoring Depth of Anaesthesia. Methods Inf. Med..

[189] Arden G.B. (1973). Recent Advances in Visual Sciences: The Visual Evoked Response in Ophthalmology.

[190] Cammann R. (1985). Use of Visual Evoked Potentials in Neurology—A Review. I. Zentralblatt Fur Neurochir..

[191] Rajbhandari Pandey K., Panday D.R., Limbu N., Shah B., Agarwal K. (2020). Effect of Smoking on Visual Evoked Potential (VEP) and Visual Reaction Time (VRT). Asian J. Med Sci..

[192] Lesiakowski P., Lubiński W., Zwierko T. (2017). Analysis of the Relationship Between Training Experience and Visual Sensory Functions in Athletes from Different Sports. Pol. J. Sport Tour..

[193] Jung T.P., Makeig S., Westerfield M., Townsend J., Courchesne E., Sejnowski T.J. (2001). Analysis and Visualization of Single-Trial Event-Related Potentials. Hum. Brain Mapp..

[194] Handy T.C. (2005). Event-Related Potentials: A Methods Handbook.

[195] Kropotov J.D. (2009). Quantitative EEG, Event-Related Potentials and Neurotherapy.

[196] Alvarenga K.F., Amorim R.B., Agostinho-Pesse R.S., Costa O.A., Nascimento L.T., Bevilacqua M.C. (2012). Speech Perception and Cortical Auditory Evoked Potentials in Cochlear Implant Users with Auditory Neuropathy Spectrum Disorders. Int. J. Pediatr. Otorhinolaryngol..

[197] Berman S., Backner Y., Krupnik R., Paul F., Petrou P., Karussis D., Levin N., Mezer A.A. (2020). Conduction Delays in the Visual Pathways of Progressive Multiple Sclerosis Patients Covary with Brain Structure. NeuroImage.

[198] Abed D.K., Almezel F., Al-Salem Y., Almuhanna D., Algharib N., Aldawood F., Albeladi Q., Kamal A., Abduljabbar O. (2020). The Correlation between the Clinical, Radiological and Visual Evoke Potential Findings in Multiple Sclerosis Patients. Bahrain Med. Bull..

[199] Ford H. (2020). Clinical Presentation and Diagnosis of Multiple Sclerosis. Clin. Med..

[200] Kook H., Gupta L., Kota S., Molfese D., Lyytinen H. (2008). An Offline/Real-Time Artifact Rejection Strategy to Improve the Classification of Multi-Channel Evoked Potentials. Pattern Recognit..

[201] Fatourechi M., Bashashati A., Ward R.K., Birch G.E. (2007). EMG and EOG Artifacts in Brain Computer Interface Systems: A Survey. Clin. Neurophysiol..

[202] Ponton C.W., Don M., Waring M.D., Eggermont J.J., Masuda A. (1993). Spatio-Temporal Source Modeling of Evoked Potentials to Acoustic and Cochlear Implant Stimulation. Electroencephalogr. Clin. Neurophysiol. Potentials Sect..

[203] Legatt A.D., Tatum W.O. (2018). Artifacts in Evoked Potential Recordings. Atlas of Artifacts in Clinical Neurophysiology.

[204] Gilley P.M., Sharma A., Dorman M., Finley C.C., Panch A.S., Martin K. (2006). Minimization of Cochlear Implant Stimulus Artifact in Cortical Auditory Evoked Potentials. Clin. Neurophysiol..

[205] Al-ani T., Cazettes F., Palfi S., Lefaucheur J.P. (2011). Automatic Removal of High-Amplitude Stimulus Artefact from Neuronal Signal Recorded in the Subthalamic Nucleus. J. Neurosci. Methods.

[206] Beer N.A.M., Velde M., Cluitmans P.J.M. (1995). Clinical Evaluation of a Method for Automatic Detection and Removal of Artifacts in Auditory Evoked Potential Monitoring. J. Clin. Monit..

[207] Chrapka P. (2018). Advances in EP and ERP Signal Processing. Ph.D. Thesis.

[208] De Bruin H., Archambeault M., Hasey G. Recording EEG During Repetitive Trans-Cranial Magnetic Stimulation. Proceedings of the International Conference on Bio-Inspired Systems and Signal Processing.

[209] Brodie B.T., Koeman H. (1981). Sample and Hold Circuit. U.S. Patent.

[210] Freeman J.A. (1971). An Electronic Stimulus Artifact Suppressor. Electroencephalogr. Clin. Neurophysiol..

[211] Roby R.J., Lettich E. (1975). A Simplified Circuit for Stimulus Artifact Suppression. Electroencephalogr. Clin. Neurophysiol..

[212] Babb T.L., Mariani E., Strain G.M., Lieb J.P., Soper H.V., Crandall P.H. (1978). A Sample and Hold Amplifier System for Stimulus Artifact Suppression. Electroencephalogr. Clin. Neurophysiol..

[213] Peper A., Grimbergen C.A. (1983). EEG Measurement During Electrical Stimulation. IEEE Trans. Biomed. Eng..

[214] Heffer L.F., Fallon J.B. (2008). A Novel Stimulus Artifact Removal Technique for High-Rate Electrical Stimulation. J. Neurosci. Methods.

[215] Schoenecker M.C., Bonham B.H. Fast Stimulus Artifact Recovery in a Multichannel Neural Recording System. Proceedings of the 2008 IEEE Biomedical Circuits and Systems Conference.

[216] Chiappa K.H., Ropper A.H. (1982). Evoked Potentials in Clinical Medicine. N. Engl. J. Med..

[217] Cao T., Wan F., Wong C., da Cruz J., Hu Y. (2014). Objective Evaluation of Fatigue by EEG Spectral Analysis in Steady-State Visual Evoked Potential-Based Brain-Computer Interfaces. BioMed. Eng. OnLine.

[218] Somers B., Francart T., Bertrand A. (2018). A Generic EEG Artifact Removal Algorithm Based on the Multi-Channel Wiener Filter. J. Neural Eng..

[219] Wang T., Özdamar Ö., Bohórquez J., Shen Q., Cheour M. (2006). Wiener Filter Deconvolution of Overlapping Evoked Potentials. J. Neurosci. Methods.

[220] Cichocki A., Gharieb R., Hoya T. Efficient Extraction of Evoked Potentials by Combination of Wiener Filtering and Subspace Methods. Proceedings of the 2001 IEEE International Conference on Acoustics, Speech, and Signal Processing (Cat. No.01CH37221).

[221] Paul J., Luft A., Hanley D., Thakor N. (2001). Coherence-Weighted Wiener Filtering of Somatosensory Evoked Potentials. IEEE Trans. Biomed. Eng..

[222] Lin B.S., Lin B.S., Chong F.C., Lai F. (2005). Adaptive Filtering of Evoked Potentials Using Higher-Order Adaptive Signal Enhancer with Genetic-Type Variable Step-Size Prefilter. Med. Biol. Eng. Comput..

[223] Ahirwal M.K., Kumar A., Singh G.K. (2014). Adaptive Filtering of EEG/ERP through Noise Cancellers Using an Improved PSO Algorithm. Swarm Evol. Comput..

[224] Dien J. (1998). Addressing Misallocation of Variance in Principal Components Analysis of Event-Related Potentials. Brain Topogr..

[225] Quian Quiroga R. (2000). Obtaining Single Stimulus Evoked Potentials with Wavelet Denoising. Phys. D Nonlinear Phenom..

[226] Quian Quiroga R., van Luijtelaar E. (2002). Habituation and Sensitization in Rat Auditory Evoked Potentials: A Single-Trial Analysis with Wavelet Denoising. Int. J. Psychophysiol..

[227] Quiroga R., Garcia H. (2003). Single-Trial Event-Related Potentials with Wavelet Denoising. Clin. Neurophysiol..

[228] Ahmadi M., Quian Quiroga R. (2013). Automatic Denoising of Single-Trial Evoked Potentials. NeuroImage.

[229] Shapiro J.M., Topiwala P.N. (2002). Embedded Image Coding Using Zerotrees of Wavelet Coefficients. Wavelet Image and Video Compression.

[230] Wang Z., Maier A., Leopold D.A., Logothetis N.K., Liang H. (2007). Single-Trial Evoked Potential Estimation Using Wavelets. Comput. Biol. Med..

[231] Iyer D., Zouridakis G. (2007). Single-Trial Evoked Potential Estimation: Comparison between Independent Component Analysis and Wavelet Denoising. Clin. Neurophysiol..

[232] Zouridakis G., Iyer D. Comparison between ICA and Wavelet-Based Denoising of Single-Trial Evoked Potentials. Proceedings of the 26th Annual International Conference of the IEEE Engineering in Medicine and Biology Society.

[233] Lee P.L., Hsieh J.C., Wu C.H., Shyu K.K., Chen S.S., Yeh T.C., Wu Y.T. (2006). The Brain Computer Interface Using Flash Visual Evoked Potential and Independent Component Analysis. Ann. Biomed. Eng..

[234] Patidar U., Zouridakis G. A Hybrid Algorithm for Artifact Rejection in EEG Recordings Based on Iterative ICA and Fuzzy Clustering. Proceedings of the 2008 30th Annual International Conference of the IEEE Engineering in Medicine and Biology Society.

[235] Palaniappan R., Ravi K. (2006). Improving Visual Evoked Potential Feature Classification for Person Recognition Using PCA and Normalization. Pattern Recognit. Lett..

[236] Palaniappan R., Anandan S., Raveendran P. Two Level PCA to Reduce Noise and EEG from Evoked Potential Signals. Proceedings of the 7th International Conference on Control, Automation, Robotics and Vision.

[237] Mowla M.R., Ng S.C., Zilany M.S.A., Paramesran R. (2016). Single-Trial Evoked Potential Estimation Using Iterative Principal Component Analysis. IEEE Sens. J..

[238] Hu L., Mouraux A., Hu Y., Iannetti G. (2010). A Novel Approach for Enhancing the Signal-to-Noise Ratio and Detecting Automatically Event-Related Potentials (ERPs) in Single Trials. NeuroImage.

[239] Zou L., Zhang Y., Yang L.T., Zhou R. (2010). Single-Trial Evoked Potentials Study by Combining Wavelet Denoising and Principal Component Analysis Methods. J. Clin. Neurophysiol..

[240] Wang D.D., Chen W., Starr P.A., de Hemptinne C., Pouratian N., Sheth S.A. (2020). Local Field Potentials and ECoG. Stereotactic and Functional Neurosurgery.

[241] Nakasatp N., Levesque M.F., Barth D.S., Baumgartner C., Rogers R.L., Sutherling W.W. (1994). Comparisons of MEG, EEG, and ECoG Source Localization in Neocortical Partial Epilepsy in Humans. Electroencephalogr. Clin. Neurophysiol..

[242] RaviPrakash H., Korostenskaja M., Castillo E.M., Lee K.H., Salinas C.M., Baumgartner J., Anwar S.M., Spampinato C., Bagci U. (2020). Deep Learning Provides Exceptional Accuracy to ECoG-Based Functional Language Mapping for Epilepsy Surgery. Front. Neurosci..

[243] Hashiguchi K., Morioka T., Yoshida F., Miyagi Y., Nagata S., Sakata A., Sasaki T. (2007). Correlation between Scalp-Recorded Electroencephalographic and Electrocorticographic Activities during Ictal Period. Seizure.

[244] Asano E., Juhasz C., Shah A., Muzik O., Chugani D.C., Shah J., Sood S., Chugani H.T. (2005). Origin and Propagation of Epileptic Spasms Delineated on Electrocorticography. Epilepsia.

[245] Logothetis N.K. (2003). The Underpinnings of the BOLD Functional Magnetic Resonance Imaging Signal. J. Neurosci..

[246] Ulbert I., Halgren E., Heit G., Karmos G. (2001). Multiple Microelectrode-Recording System for Human Intracortical Applications. J. Neurosci. Methods.

[247] van’t Klooster M.A., van Klink N.E., Zweiphenning W.J., Leijten F.S., Zelmann R., Ferrier C.H., van Rijen P.C., Otte W.M., Braun K.P., Huiskamp G.J. (2017). Tailoring Epilepsy Surgery with Fast Ripples in the Intraoperative Electrocorticogram: Tailoring Epilepsy Surgery With Fast Ripples. Ann. Neurol..

[248] Sellers K.K., Schuerman W.L., Dawes H.E., Chang E.F., Leonard M.K. Comparison of Common Artifact Rejection Methods Applied to Direct Cortical and Peripheral Stimulation in Human ECoG. Proceedings of the 2019 9th International IEEE/EMBS Conference on Neural Engineering (NER).

[249] Caldwell D.J., Cronin J.A., Rao R.P.N., Collins K.L., Weaver K.E., Ko A.L., Ojemann J.G., Kutz J.N., Brunton B.W. (2020). Signal Recovery from Stimulation Artifacts in Intracranial Recordings with Dictionary Learning. J. Neural Eng..

[250] Schweigmann M., Koch K.P., Auler F., Kirchhoff F. (2018). Improving Electrocorticograms of Awake and Anaesthetized Mice Using Wavelet Denoising. Curr. Dir. Biomed. Eng..

[251] Freeman W.J., Rogers L.J., Holmes M.D., Silbergeld D.L. (2000). Spatial Spectral Analysis of Human Electrocorticograms Including the Alpha and Gamma Bands. J. Neurosci. Methods.

[252] Deeb S.E. (2019). Analysis of Globus Pallidus Local Field Potentials and Electrocorticograms of Patients Diagnosed with Parkinson’s Disease—ProQuest. Ph.D. Thesis.

[253] Chen Z., Huang L., Shen Y., Wang J., Zhao R., Dai J. A New Algorithm for Classification of Ictal and Pre-Ictal Epilepsy ECoG Using MI and SVM. Proceedings of the 2017 International Conference on Signals and Systems (ICSigSys).

[254] Hossain G., Myers M.H., Kozma R., Hutchison D., Kanade T., Kittler J., Kleinberg J.M., Mattern F., Mitchell J.C., Naor M., Nierstrasz O., Pandu Rangan C., Steffen B. (2012). Study of Phase Relationships in ECoG Signals Using Hilbert-Huang Transforms. Advances in Brain Inspired Cognitive Systems.

[255] Seo J.H., Tsuda I., Lee Y.J., Ikeda A., Matsuhashi M., Matsumoto R., Kikuchi T., Kang H. (2020). Pattern Recognition in Epileptic EEG Signals via Dynamic Mode Decomposition. Mathematics.

[256] Ince N.F., Goksu F., Tewfik A.H. An ECoG Based Brain Computer Interface with Spatially Adapted Time-Frequency Patterns. Proceedings of the 2008 International Conference on Bio-Inspired Systems and Signal Processing (BIOSIGNALS 2008).

[257] Herff C., Krusienski D.J., Kubben P. (2020). The potential of stereotactic-EEG for brain-computer interfaces: Current progress and future directions. Front. Neurosci..

[258] Guenot M., Isnard J., Ryvlin P., Fischer C., Ostrowsky K., Mauguiere F., Sindou M. (2001). Neurophysiological monitoring for epilepsy surgery: The Talairach SEEG method. Stereotact. Funct. Neurosurg..

[259] Bartolomei F., Lagarde S., Wendling F., McGonigal A., Jirsa V., Guye M., Bénar C. (2017). Defining epileptogenic networks: Contribution of SEEG and signal analysis. Epilepsia.

[260] Mullin J.P., Shriver M., Alomar S., Najm I., Bulacio J., Chauvel P., Gonzalez-Martinez J. (2016). Is SEEG safe? A systematic review and meta-analysis of stereo-electroencephalography—Related complications. Epilepsia.

[261] Gordon E., Konopka L.M. (2005). EEG databases in research and clinical practice: Current status and future directions. Clin. EEG Neurosci..

[262] Agarwal M. EEG Databases—GitHub Repository. 2021. https://github.com/meagmohit/EEG-Datasets.

[263] Van Horn J.D., Grafton S.T., Rockmore D., Gazzaniga M.S. (2004). Sharing neuroimaging studies of human cognition. Nat. Neurosci..

[264] Physionet—Data Base. 2021. https://physionet.org.

[265] Goldberger A.L., Amaral L.A.N., Glass L., Hausdorff J.M., Ivanov P.C., Mark R.G., Mietus J.E., Moody G.B., Peng C.K., Stanley H.E. (2000). PhysioBank, PhysioToolkit, and PhysioNet: Components of a New Research Resource for Complex Physiologic Signals. Circulation.

[266] Sweeney K.T., Ayaz H., Ward T.E., Izzetoglu M., McLoone S.F., Onaral B. (2012). A methodology for validating artifact removal techniques for physiological signals. IEEE Trans. Inf. Technol. Biomed..

[267] Kemp B., Zwinderman A.H., Tuk B., Kamphuisen H.A., Oberye J.J. (2000). Analysis of a sleep-dependent neuronal feedback loop: The slow-wave microcontinuity of the EEG. IEEE Trans. Biomed. Eng..

[268] Abel J.H., Badgeley M.A., Meschede-Krasa B., Schamberg G., Garwood I.C., Lecamwasam K., Chakravarty S., Zhou D.W., Keating M., Purdon P.L. (2021). Machine learning of EEG spectra classifies unconsciousness during GABAergic anesthesia. PLoS ONE.

[269] Detti P., Vatti G., Zabalo Manrique de Lara G. (2020). EEG Synchronization Analysis for Seizure Prediction: A Study on Data of Noninvasive Recordings. Processes.

[270] Detti P. (2020). Siena Scalp EEG Database (Version 1.0.0). PhysioNet.

[271] Shoeb A.H. (2009). Application of Machine Learning to Epileptic Seizure Onset Detection and Treatment. Ph.D. Thesis.

[272] Schalk G., McFarland D.J., Hinterberger T., Birbaumer N., Wolpaw J.R. (2004). BCI2000: A general-purpose brain-computer interface (BCI) system. IEEE Trans. Biomed. Eng..

[273] Zyma I., Tukaev S., Seleznov I., Kiyono K., Popov A., Chernykh M., Shpenkov O. (2019). Electroencephalograms during mental arithmetic task performance. Data.

[274] Matran-Fernandez A., Poli R. (2017). Towards the automated localisation of targets in rapid image-sifting by collaborative brain-computer interfaces. PLoS ONE.

[275] Oikonomou V.P., Liaros G., Georgiadis K., Chatzilari E., Adam K., Nikolopoulos S., Kompatsiaris I. (2016). Comparative evaluation of state-of-the-art algorithms for SSVEP-based BCIs. arXiv.

[276] Hu L., Zhang Z. (2019). EEG Signal Processing and Feature Extraction.

[277] Chen S., He Z., Han X., He X., Li R., Zhu H., Zhao D., Dai C., Zhang Y., Lu Z. (2019). How big data and high-performance computing drive brain science. Genom. Proteom. Bioinform..

[278] Landhuis E. (2017). Neuroscience: Big brain, big data. Nature.

[279] Cavanagh J.F. (2019). Electrophysiology as a theoretical and methodological hub for the neural sciences. Psychophysiology.

[280] Khosla A., Khandnor P., Chand T. (2020). A comparative analysis of signal processing and classification methods for different applications based on EEG signals. Biocybern. Biomed. Eng..

[281] Blankertz B., Lemm S., Treder M., Haufe S., Müller K.R. (2011). Single-trial analysis and classification of ERP components—A tutorial. NeuroImage.

[282] Makeig S., Kothe C., Mullen T., Bigdely-Shamlo N., Zhang Z., Kreutz-Delgado K. (2012). Evolving signal processing for brain–computer interfaces. Proc. IEEE.

[283] Müller K.R., Tangermann M., Dornhege G., Krauledat M., Curio G., Blankertz B. (2008). Machine learning for real-time single-trial EEG-analysis: From brain–computer interfacing to mental state monitoring. J. Neurosci. Methods.

[284] Neuner I., Arrubla J., Werner C.J., Hitz K., Boers F., Kawohl W., Shah N.J. (2014). The default mode network and EEG regional spectral power: A simultaneous fMRI-EEG study. PLoS ONE.

[285] Ritter P., Villringer A. (2006). simultaneous EEG–fMRI. Neurosci. Biobehav. Rev..

[286] Huster R.J., Debener S., Eichele T., Herrmann C.S. (2012). Methods for simultaneous EEG-fMRI: An introductory review. J. Neurosci..

[287] Mishra V., Gautier N.M., Glasscock E. (2018). Simultaneous Video-EEG-ECG monitoring to identify neurocardiac dysfunction in mouse models of epilepsy. J. Vis. Exp. Jove.

[288] Niegowski M., Zivanovic M. (2014). ECG-EMG separation by using enhanced non-negative matrix factorization. Proceedings of the 2014 36th Annual International Conference of the IEEE Engineering in Medicine and Biology Society.

[289] Haddix C., Bahrani A.A., Kawala-Janik A., Besio W.G., Yu G., Sunderam S. (2017). Trial measurement of movement-related cortical dynamics using electroencephalography and diffuse correlation spectroscopy. Proceedings of the 2017 22nd International Conference on Methods and Models in Automation and Robotics (MMAR).

[290] Yang J., Cha S., Yun D., An J. (2020). Probe Configuration Design for Closed-loop Multi-Channel fNIRS-tDCS BCI. Proceedings of the 2020 8th International Winter Conference on Brain-Computer Interface (BCI).

[291] Matarasso A.K., Rieke J.D., White K., Yusufali M.M., Daly J.J. (2021). Combined real-time fMRI and real time fNIRS brain computer interface (BCI): Training of volitional wrist extension after stroke, a case series pilot study. PLoS ONE.

[292] Val-Calvo M., Álvarez-Sánchez J.R., Ferrández-Vicente J.M., Díaz-Morcillo A., Fernández-Jover E. (2020). Real-time multi-modal estimation of dynamically evoked emotions using EEG, heart rate and galvanic skin response. Int. J. Neural Syst..

[293] Park H.J., Han J.M., Jeong D.U., Park K.S. (1998). A study on the elimination of the ECG artifact in the polysomnographic EEG and EOG using AR model. Proceedings of the 20th Annual International Conference of the IEEE Engineering in Medicine and Biology Society. Vol. 20 Biomedical Engineering Towards the Year 2000 and Beyond (Cat. No. 98CH36286).

[294] Sanei S., Chambers J.A. (2013). EEG Signal Processing.

[295] Yan W.X., Mullinger K.J., Brookes M.J., Bowtell R. (2009). Understanding gradient artefacts in simultaneous EEG/fMRI. Neuroimage.

[296] Guarnieri R. (2021). Applications. Ph.D. Thesis.

[297] Rogasch N.C., Thomson R.H., Farzan F., Fitzgibbon B.M., Bailey N.W., Hernandez-Pavon J.C., Daskalakis Z.J., Fitzgerald P.B. (2014). Removing artefacts from TMS-EEG recordings using independent component analysis: Importance for assessing prefrontal and motor cortex network properties. Neuroimage.

[298] Zander T.O., Kothe C., Jatzev S., Gaertner M. (2010). Enhancing human-computer interaction with input from active and passive brain-computer interfaces. Brain-Computer Interfaces.

[299] Bhattacharyya S., Das S., Das A., Dey R., Dhar R. (2021). Neuro-feedback system for real-time BCI decision prediction. Microsyst. Technol..

[300] Martišius I., Damaševičius R. (2016). A prototype SSVEP based real time BCI gaming system. Comput. Intell. Neurosci..

[301] Martinek R., Ladrova M., Sidikova M., Jaros R., Behbehani K., Kahankova R., Kawala-Sterniuk A. (2021). Advanced Bioelectrical Signal Processing Methods: Past, Present and Future Approach—Part I: Cardiac Signals. Sensors.

[302] Alipour A., Rezai A., Hashemi T., Yousefpour N. (2017). The effectiveness of cognitive behavioral therapy focused on lifestyle modification to increase monitoring vital signs and coronary heart disease and psychological well-being. Q. J. Health Psychol..

[303] Fioranelli F., Le Kernec J., Shah S.A. (2019). Radar for health care: Recognizing human activities and monitoring vital signs. IEEE Potentials.

